# Molecular mechanisms underpinning quantitative resistance to *Phytophthora sojae* in *Glycine max* using a systems genomics approach

**DOI:** 10.3389/fpls.2023.1277585

**Published:** 2023-11-07

**Authors:** Cassidy R. Million, Saranga Wijeratne, Stephanie Karhoff, Bryan J. Cassone, Leah K. McHale, Anne E. Dorrance

**Affiliations:** ^1^ Department of Plant Pathology, The Ohio State University, Wooster, OH, United States; ^2^ Center for Soybean Research and Center for Applied Plant Sciences, The Ohio State University, Columbus, OH, United States; ^3^ Molecular and Cellular Imaging Center, The Ohio State University, Wooster, OH, United States; ^4^ Translational Plant Sciences Graduate Program, The Ohio State University, Columbus, OH, United States; ^5^ Department of Biology, Brandon University, Brandon, Manitoba, MB, Canada; ^6^ Department of Horticulture and Crop Science, The Ohio State University, Columbus, OH, United States

**Keywords:** Glycine max, soybean, *Phytophthora sojae*, eQTL, systems genomics, master regulators, weighted gene co-expression network analysis

## Abstract

Expression of quantitative disease resistance in many host–pathogen systems is controlled by genes at multiple loci, each contributing a small effect to the overall response. We used a systems genomics approach to study the molecular underpinnings of quantitative disease resistance in the soybean-*Phytophthora sojae* pathosystem, incorporating expression quantitative trait loci (eQTL) mapping and gene co-expression network analysis to identify the genes putatively regulating transcriptional changes in response to inoculation. These findings were compared to previously mapped phenotypic (phQTL) to identify the molecular mechanisms contributing to the expression of this resistance. A subset of 93 recombinant inbred lines (RILs) from a Conrad × Sloan population were inoculated with *P. sojae* isolate 1.S.1.1 using the tray-test method; RNA was extracted, sequenced, and the normalized read counts were genetically mapped from tissue collected at the inoculation site 24 h after inoculation from both mock and inoculated samples. In total, more than 100,000 eQTLs were mapped. There was a switch from predominantly *cis*-eQTLs in the mock treatment to an almost entirely nonoverlapping set of predominantly *trans*-eQTLs in the inoculated treatment, where greater than 100-fold more eQTLs were mapped relative to mock, indicating vast transcriptional reprogramming due to *P. sojae* infection occurred. The eQTLs were organized into 36 hotspots, with the four largest hotspots from the inoculated treatment corresponding to more than 70% of the eQTLs, each enriched for genes within plant–pathogen interaction pathways. Genetic regulation of *trans*-eQTLs in response to the pathogen was predicted to occur through transcription factors and signaling molecules involved in plant–pathogen interactions, plant hormone signal transduction, and MAPK pathways. Network analysis identified three co-expression modules that were correlated with susceptibility to *P. sojae* and associated with three eQTL hotspots. Among the eQTLs co-localized with phQTLs, two *cis*-eQTLs with putative functions in the regulation of root architecture or jasmonic acid, as well as the putative master regulators of an eQTL hotspot nearby a phQTL, represent candidates potentially underpinning the molecular control of these phQTLs for resistance.

## 1 Introduction

Quantitative disease resistance (QDR) is a type of host resistance that generally involves multiple loci acting additively, each with a small to moderate overall effect on limiting disease development (Young, 1996; Poland et al., 2009; St. Clair, 2010; Roux et al., 2014; Niks et al., 2015; French et al., 2016; Corwin and Kliebenstein, 2017; Nelson et al., 2018). Numerous phenotypic quantitative trait loci (phQTL) (Jansen et al., 2009; Acharjee et al., 2018) for disease resistance have been mapped in soybean (Lin et al., 2022) to biotrophic pathogens, such as soybean cyst nematode (SCN: *Heterodera glycine*) (Wu et al., 2009) and powdery mildew (*Microsphaera diffusa*) (Jun et al., 2012); hemibiotrophic pathogens *Phytophthora sojae* (Burnham et al., 2003; Weng et al., 2007; Han et al., 2008; Li et al., 2010; Tucker et al., 2010; Wang et al., 2010; Wu et al., 2011; Nguyen et al., 2012; Wang et al., 2012b; Lee et al., 2013a; Lee et al., 2013b; Lee et al., 2014; Abeysekara et al., 2016; Stasko et al., 2016) and *Phialophora gregata* (Rincker et al., 2016); and necrotrophic pathogens *Sclerotinia sclerotiorum* (Kim and Diers, 2000; Arahana et al., 2001; Guo et al., 2008; Vuong et al., 2008; Zhao et al., 2015). However, the causal genes for QDR have only been identified and verified in the SCN soybean pathosystem through genetic mapping, gene silencing, and complementation experiments (Cook et al., 2012; Cook et al., 2014; Bayless et al., 2018). Impediments to the discovery of causal genes for QDR are often attributed to the intricate biology of plant-pathogen interactions (Zhou et al., 2009; Corwin et al., 2016; Corwin and Kliebenstein, 2017; Nelson et al., 2018). Additionally, the methods to functionally evaluate candidate genes underlying specific phQTLs are difficult, as each phQTL has a relatively small effect on the final phenotype, and modification of a single gene often yields inconclusive results (Salvi and Tuberosa, 2005; Poland et al., 2009; Corwin and Kliebenstein, 2017). Receptor-like kinases (RLKs) and nucleotide-binding leucine-rich repeat proteins (NLRs) are recognized as canonical resistance proteins (Niks et al., 2015; French et al., 2016; Pilet-Nayel et al., 2017); however, these proteins, which commonly function in qualitative or *R*-gene-mediated defense, only account for a small portion of genes involved in QDR to date (Poland et al., 2009; Corwin et al., 2016; Nelson et al., 2018). QDR has been hypothesized to be controlled by proteins with a wide range of functions, and that has, thus far, been born out through functional studies (Poland et al., 2009; St. Clair, 2010; Roux et al., 2014; Niks et al., 2015; French et al., 2016; Corwin and Kliebenstein, 2017). For example, genes with functions more commonly associated with plant development, cell wall reinforcement, RNA processing, and defense compounds were associated with QDR (Poland et al., 2009; Corwin et al., 2016; Corwin and Kliebenstein, 2017; Nelson et al., 2018). This functional diversity of genes involved in QDR makes it difficult to predict causal candidate genes based solely on homology-predicted functions and gene positions relative to phQTL.

Due to the adaptation of *P. sojae* populations to *R*-gene-mediated resistance in soybean, QDR is preferred in some growing regions to manage Phytophthora root and stem rot (PRR) (Grau et al., 2004; Dorrance et al., 2009; Schmitthenner, 1985). In this plant-pathogen system, QDR is a partial resistance that allows for some pathogen growth and reproduction and generally consists of several loci, each contributing a minor effect or one major effect locus combined with several minor effect loci (Tooley and Grau, 1982; Mideros et al., 2007; Weng et al., 2007; Han et al., 2008; Li et al., 2010; Tucker et al., 2010; Wu et al., 2011; Nguyen et al., 2012; Wang et al., 2012a; Wang et al., 2012b; Lee et al., 2013a; Lee et al., 2013b; Lee et al., 2014; Abeysekara et al., 2016; Stasko et al., 2016; Scott et al., 2019; de Ronne et al., 2022). Nine different parental combinations have produced numerous phQTLs contributing towards *P. sojae* quantitative resistance in soybean (Burnham et al., 2003; Weng et al., 2007; Han et al., 2008; Li et al., 2010; Tucker et al., 2010; Wang et al., 2010; Wu et al., 2011; Nguyen et al., 2012; Wang et al., 2012a; Wang et al., 2012b; Lee et al., 2013a; Lee et al., 2013b; Lee et al., 2014; Abeysekara et al., 2016; Stasko et al., 2016). Of these, 60 phQTLs, most of small effect, were mapped in multiple generations of a ‘Conrad’ × ‘Sloan’ recombinant inbred line (RIL) population using different field, greenhouse, and lab screening methodologies to collect the phenotypic data (Burnham et al., 2003; Weng et al., 2007; Han et al., 2008; Li et al., 2010; Wang et al., 2010; Wu et al., 2011; Wang et al., 2012b; Stasko et al., 2016). Each of these phQTLs encompasses large regions of the chromosome, which contain many genes (Wang et al., 2012b; Stasko et al., 2016), making it difficult to identify the causal genes based on position alone.

Few studies have examined the physiological and molecular mechanisms of quantitative resistance in soybean to *P. sojae*; however, all have concluded that this is a complex trait. Although only a few mechanisms for QDR have been substantiated in plant systems (Nelson et al., 2018), numerous hypotheses have been developed for resistance to *P. sojae* in soybeans from several previous studies. These hypotheses include plant hormone signal transduction, including auxin acting as a susceptibility factor (Wang et al., 2010; Wang et al., 2012b; Stasko et al., 2020); suberin playing a role in slowing *P. sojae* hyphal infection in epidermal walls and middle lamellae (Thomas et al., 2007; Ranathunge et al., 2008); and the phenylpropanoid pathway acting as a positive regulator of *P. sojae* infection by increased content of glyceollin, daidzein, genistein, and salicylic acid (SA) (Abbasi et al., 2001; Mohr and Cahill, 2001; Graham et al., 2003; Lygin et al., 2013; Zhang et al., 2017). Components of the isoflavonoid pathway have been implicated in acting as antioxidants to reduce reactive oxygen species (ROS) and enhance QDR to *P. sojae* (Xu et al., 2012; Wong et al., 2014; Cheng et al., 2015; Dastmalchi et al., 2017). QDR in *P. sojae* has also been associated with increased expression of genes coding for pathogenesis-related 1a protein (PR1a), matrix metalloproteinases, basic peroxidases, and β-1,3-endoglucanases (Vega-Sánchez et al., 2005), as well as ubiquitination, plant cell structural modifications, serine-threonine kinase, and basal resistance (Wang et al., 2012b; Karhoff et al., 2022). Recently, a gene annotated as a major latex protein expressed in the roots and associated with biotic stress was implicated in QDR (de Ronne et al., 2022). In addition to these pathways, there are other well-documented pathways involved in plant defense against pathogens, including mitogen-activated protein kinase (MAPK) cascades and plant-pathogen interaction pathways (e.g., pathogen-associated molecular patterns (PAMP) and effector-triggered immunity (ETI) pathways), which may also play a role in QDR (Ausubel, 2005; Glazebrook, 2005; Chisholm et al., 2006; Jones and Dangl, 2006; Boller and Felix, 2009; Dodds and Rathjen, 2010; Gassmann and Bhattacharjee, 2012; Spoel and Dong, 2012; Zipfel, 2014; Cui et al., 2015; Nelson et al., 2018). Taken together, these reports emphasize the complexity of QDR mechanisms and responses to infection. Zhou et al. (2009) showed that by 5 days postinoculation with *P. sojae*, from tissue collected in front of the advancing lesion, 97% of the genes in the soybean genome responded to infection or genetic variation based on microarray data. Amid this genome-wide transcriptional reprogramming, it is difficult to identify the specific causal mechanisms, pathways, and putative candidate genes. Thus, a more robust approach is needed to elucidate the molecular mechanisms underlying QDR.

Numerous studies in plant-pathogen interactions have utilized a systems genomics approach to identify the molecular mechanisms that are controlled by a complex of genes, pathways, and networks contributing to the overall expression of a phenotype (Jansen and Nap, 2001; Kliebenstein, 2009; Druka et al., 2010; Feltus, 2014). This approach maps both phQTLs and expression (eQTLs) and combines these data with a gene network analysis to identify the specific alleles that control or contribute to the overall expression of resistance during a plant–pathogen interaction (Kliebenstein, 2009; Druka et al., 2010; Feltus, 2014). Using this approach, advancements were made towards understanding the mechanisms of QDR resistance in barley (*Hordeum vulgare* L.) to *Puccinia hordei* (Druka et al., 2008; Chen et al., 2010) and in maize (*Zea mays* L.) to *Cercospora zeina* (Christie et al., 2017). In the barley-*Puccinia hordei* system, the total number of candidate genes underlying phQTLs was reduced at four different loci, with the identification of a histidine kinase as a novel resistance gene at one locus (Druka et al., 2008). In a later study, the total number of candidate genes for QDR to *Puccinia hordei* was reduced to six candidates for barley *Rphq11* (Chen et al., 2010). Co-expression of *coronatine-insensitive 1* (*COI1*) and jasmonate responses in maize were correlated with resistance to *C. zeina*, while pathogen manipulation of the host plant through the diterpenoid biosynthesis pathway was associated with susceptibility (Christie et al., 2017).

Due to the nature of the *P. sojae*-soybean pathosystem response, in which a small proportion of the total phenotype is contributed by each locus and a large number of loci encompassing numerous genes respond to *P. sojae* infection, we have taken a systems genomics approach to elucidate the molecular mechanisms of QDR in soybean toward *P. sojae*. In this study, we aimed to (1) understand the transcriptional reprogramming that occurs earlier in the infection process during the transition from biotrophic to necrotrophic between the well-studied soybean cultivars, Conrad, which has high levels of QDR, and Sloan, which is moderately susceptible; (2) map the genetic control of the differential transcriptional response to inoculation with *P. sojae*; (3) identify functional enrichment of genes within eQTL hotspots and co-expression modules associated with disease; and (4) identify candidate genes. The emphasis in this study is placed on identifying factors that elicit expression of quantitative resistance and not those expressed during the *R*-gene (*Rps* gene) response, which have also been recently studied using a transcriptomic approach (Lin et al., 2014; Hale et al., 2023a).

Three types of analyses were used, including phQTL mapping, eQTL mapping, and weighted gene co-expression analysis (WGCNA). The integration of the three analyses allowed for a greater understanding of the relationships between gene expression and the resulting disease phenotypes. Ultimately, using the expression data of 93 RILs during the early stages of the infection process [24 hours after inoculation (hai)] as the switch from biotrophy to necrotrophy is occurring (Moy et al., 2004), we were able to map more than 100,000 eQTLs, identifying co-regulated gene modules associated with disease and five putative candidate genes for three phQTLs. Putative master regulators were identified for 16 key eQTL hotspots. This study is the first to our knowledge to elucidate the specific candidate genes that may regulate the extensive changes that occur during transcriptional reprogramming due to pathogen infection by utilizing eQTL mapping in inoculated and non-inoculated tissues within the same population.

## 2 Materials and methods

### 2.1 Phenotyping and RNA extraction

A subset of 93 F_9:11_ RILs from the full population of 316 RILs derived from a cross between the cultivars Conrad (resistant) and Sloan (susceptible) were selected randomly for the eQTL study. The parents and RILs were inoculated with *P. sojae* 1.S.1.1 as previously described (Wang et al., 2012b). Briefly, the roots of 7-day-old plants that were grown for one week in 29.5-ml Styrofoam cups containing vermiculite (Perlite Vermiculite Packing Industries, Inc., North Bloomfield, OH, USA) were washed in tap water. Seedlings were placed on a plastic tray on top of a cotton wicking pad and a polyester cloth. A wound on the main tap root of each plant was made with a scalpel approximately 2.5 cm below the crown and covered with a mycelial slurry of seven-day-old 1.S.1.1 of *P. sojae* grown on lima bean (*Phaseolus lunatus* L.) agar. These trays were placed in buckets and kept in a growth chamber at 25°C, 20% relative humidity (RH), on a 14-h light:10-h dark cycle. The experimental design was a randomized incomplete block design with three replications with each block containing 50–100 RILs. There were three trays for each RIL in each block. The first two trays were for RNA isolation where each tray was either inoculated with 1.S.1.1 or mock inoculated with lima bean agar (without *P. sojae*). No RNA was isolated from the third tray which was inoculated with 1.S.1.1, and the lesion length was measured 7 days after inoculation (dai) from the top of the inoculation site to the leading edge of the lesion margin to ensure that inoculations had been successful. A schematic for the experimental design is shown in **Supplementary Figure S1**.

All agar was gently removed using a Kimwipe, and a 1-cm section of tissue from the inoculation site was collected 24 hai from the mock and inoculated trays. Tissue from eight to 10 plants from a single RIL was collected, and tissue from all three replicates of each RIL was pooled for RNA extraction for a total of ~24–30 plants per RIL. Tissue was ground in liquid nitrogen, and RNA was extracted using a Qiagen RNeasy Plant Mini Kit (Hilden, Germany) following the manufacturer’s protocols.

RNA was quality and quantity checked using the TapeStation (Agilent Technologies, Santa Clara, CA, USA) and Qubit 2.0 Fluorometer (Invitrogen, Carlsbad, CA, USA), respectively. RNA quality and quantity standards were met for both the mock-inoculated and inoculated treatment for each of the 93 RILs. Sequence data from this study can be found in the NCBI BioProject: PRJNA478334.

### 2.2 Genotypic and phenotypic data

Phenotypic QTLs have been previously reported in the Conrad × Sloan recombinant inbred population of 316 individuals (Stasko et al., 2016), a subset of which was used for this study. The methods for inoculation, experimental design, and statistical analysis for mapping were described in greater detail (Wang et al., 2010; Wang et al., 2012b; Stasko et al., 2016). To construct a genetic map to carry out both eQTL and phQTL mapping, genotypic data was obtained from Stasko et al. (2016). While included in WGCNA, RIL 12280 was removed from the phQTL and eQTL analyses due to missing genotypic data. The normality of the data was evaluated using the Shapiro–Wilk test and visually assessed by histograms and QQ plots.

To account for environment variation, best linear unbiased predictor (BLUP) values for RILs were extracted from the model by adjusting mean lesion lengths of 92 RILs to the checks (cultivars OX20-8 (*Rps1a*, no partial resistance), Williams 82 (*Rps1k*, moderate partial resistance), Conrad (parent, high partial resistance), Sloan (parent, moderately susceptible)) and removing environmental variation in R version 3.5.1 (R Core Team, 2018) with the package -’lme4’ using the lmer function in version 1.1-19 (Bates et al., 2014). The model applied was *Y_ijk_ = µ + R_i_ + T_j_ + G*(*C*)*_jk_ + ϵ_ijk_* where *µ* = overall mean, *R_i_* = effect of the *i*th replication, *T_j_* = effect of the *j*th type of entry, *G*(*C*)*_jk_* = effect of the *k*th genotype within the type for RIL only, and *ϵ_ijk_* = experimental error. The RILs in this study represent a subset of all possible genetic combinations and were treated as random (Green and Tukey, 1960). The same principle was applied to the random effect of replication, while the assigned category of type of entry (a category for RILs as well as each of the checks) was treated as fixed. Variance components were estimated using the maximum likelihood method. Composite interval mapping (R/qtl; Broman et al., 2003) was utilized to confirm phQTL peaks that were associated with lesion length BLUP values in the 316 RIL population from Stasko et al. (2016).

### 2.3 RNA sequencing

For each of the 184 samples consisting of ~24–30 plants per RIL per treatment, 1 µg of total RNA was converted to complementary DNA libraries (Molecular and Cellular Imaging Center, Ohio Agricultural Research and Development Center) and sequenced using Illumina HiSeq paired-end (PE) 100 base-pair reads. The average reads per sample was 3,126,863, ranging from 2,484,193 to 5,087,094 reads per sample. While only a single sample was sequenced for each RIL:treatment combination, the use of a highly inbred RIL population makes it such that each allele is represented approximately 46 times per treatment (50% of 92) and therefore provides replication within the genome-wide analyses. In brief, all reads were quality checked, adapter removed, and quality trimmed using bioinformatics tools FastQC (Andrews, 2010) and BBTools (BBMap; Bushnell, 2014). All reads were mapped to the reference genome (*Glycine max* Wm82.a2.v1) retrieved from Phytozome^1^, using CLCBio (CLC Genomics Workbench 9.5.3^2^. Reads mapped to the reference genome were counted using FeatureCounts (Liao et al., 2014). Data were normalized using edgeR (Robinson et al., 2010) using the trimmed mean of the M-values (TMM) normalization.

### 2.4 iBMQ eQTL mapping

The integrated hierarchical Bayesian model for multivariate eQTL mapping (iBMQ; Imholte et al., 2013) is a multivariate mapping method that implements a multi-loci approach to allow for the identification of complex traits that are being controlled by multiple genes. iBMQ parameters are estimated using a Markov Chain Monte Carlo (MCMC) algorithm that models concurrently all genes and SNPs. The current version of iBMQ executed is available online^3^. Normalized unfiltered expression data (RNA-seq data) and genotyping data, including SNPs that were identified in the newly conducted linkage map for all RILs, were used as inputs into iBMQ. These data sets were formatted into an *ExpressionSet* and *Snpset* using Biobase in Bioconductor 3.7 (Huber et al., 2015). The eQTLs were mapped by using gene expression (e-trait) as a phenotypic trait identifying the SNP association for mock and inoculated treatments and mapped separately with 100,000 iterations with a burn-in of 50,000 to produce a posterior probability of association (PPA). A false discovery rate (FDR) of 10% was used to determine the PPA significance cutoff (Imholte et al., 2013).

Significantly mapped eQTLs were classified as either *cis*- or *trans*- using the eqtlClassifier in the iBMQ package. Classification of *cis*- or *trans*-eQTLs was determined by physical position; if a SNP was within 5 Mb of the gene it controlled, it was classified as *cis*-, and if a SNP was farther than 5Mb from the gene controlled, it was classified as *trans*-. The physical positions of genes and SNPs were determined using the reference genome annotation, Williams 82 v.a2.v1^4^.

The function *hotspot finder* inside the iBMQ package was used to identify individual markers (SNPs) that are associated with the expression of several genes and indicate eQTL, hotspots as described by Imholte et al. (2013). A SNP was identified as a hotspot if it was associated with >20 genes.

### 2.5 Gene enrichment

Significant *cis-* and *trans*-eQTLs, gene suites at eQTL hotspots, and co-expression modules were subjected to GO enrichment. AgriGO (v2.0; Tian et al., 2017) was used for GO enrichment analysis. Targeted QDR pathway mechanisms were also explored by extracting genes via KEGG (Kanehisa and Goto, 2000 ^5^) from NCBI BioSystems^6^. Gene models from the reference soybean genome that met read-mapping thresholds for inclusion in eQTL mapping were used as the reference set for enrichment analyses (Schmutz et al., 2010). Fisher’s exact test (Fisher, 1935) and multitest Hochberg FDR adjustment (Benjamini and Hochberg, 1995) were used to determine significance.

### 2.6 Weighted gene co-expression analysis

WGCNA was carried out according to Langfelder and Horvath (2008) using modified R-scripts (WGCNA **Methods M1**). Prior to WGCNA analysis, RNA-sequencing counts from 92 RILs plus the parents of the population were normalized using R Bioconductor package edgeR (Robinson et al., 2010). First trimmed means of *M*-values (TMM) were used to calculate normalization factors for all sample counts and were subjected to a log2 transformation. Genes were filtered by the threshold of greater than 2 counts per million (cpm) across 50 samples. Ultimately, 27,666 genes were used in the input matrix. Due to sample count bias, a consensus approach was taken. Based on the smallest standard deviation representing susceptible and resistant individuals separately, 30 resistant and 30 susceptible individuals were selected, and the minimum standard deviation was determined (10,000 iterations) and used as a cutoff to select the subsample of 30 resistant and 30 susceptible individuals. The process of subsampling individuals who met the standard deviation cutoff through generating modules was done in 20,000 iterations and generated for 20 network analyses.

To examine the physiological relevance of each module within the network analyses, phenotypic traits were correlated to the module genes’ expression using log expression eigengene values for each module regressed against the lesion length values of the RILs. Consensus networks were constructed independently for the positively and negatively correlated modules and again related to phenotypic traits (WGCNA **Methods M2**).

To determine the driving factors of co-expression modules, modules that were significantly correlated to PRR disease (*p*-value ≤ 0.05) were also correlated to inoculated eQTL hotspots, adapting methods from Zhang and Horvath (2005) to integrate SNP-based significance (PPA of eQTL) with network properties (gene significance) of co-expression module (WGCNA **Methods M3**).

### 2.7 Co-localization of eQTLs with phQTLs

Co-localization of *cis*-eQTLs with phQTLs was based on the physical coordinates of the *cis*-eQTLs gene model being within the flanking marker positions for selected phQTLs reported from the present and previous studies (**Table 1**). All phQTLs that could not be positioned onto the genome based on marker positions were removed from the data set, for a total of six out of 28 phQTLs. Based on the physical coordinates of markers flanking phQTLs, overlapping phQTLs were consolidated. To determine if the number of co-localized *cis*-eQTLs and consolidated phQTLs was significantly greater than expected, *cis*-eQTLs were permuted across all gene models eligible for eQTL analysis. A threshold (*α* = 0.05) was set based on 1,000 permutations.

**Table 1:** Co-localization of *cis-* and *trans-*eQTL as well as hotpots with consolidated phenotypic quantitative trait loci (phQTLs) mapped in the sub-population and previous phQTL mapped in multiple generations of the Conrad × Sloan RIL population.

phQTL ID	Left marker-right marker	Physical position^a^	*Cis*-eQTL gene	*Trans*-eQTL gene or hotspot (marker)
phQTL_1^b,c^	ss715583994-ss715582762	1: 49814688-51043150	*Glyma.01G160600*	*Glyma.10G026500* (ss715579958)
			*Glyma.01G162600*	
			*Glyma.01G170600*	*Glyma.10G026500* (ss715579975)
			*Glyma.01G171300*	
phQTL_4^c^	ss715588277-ss715588347	4: 46096228-46536196	NA	NA
phQTL_9^c^	ss715603084	9: 15487393-19208849	NA	NA
phQTL_16^c^	ss715624395-ss715624634	16: 3124736-3362395	NA	NA
phQTL_18a^b,d^	ss715582789-BARCSOYSSR_18_1710	18: 53019336-53902882	NA	NA
phQTL_18b^b–e^	BARCSOYSSR_18_1777-BARCSOYSSR_18_1949	18: 54744147-57972957	*Glyma.18G270900*	*Glyma.08G249200* (ss715632217)
phQTL_19a^b^	ss715582079-BARCSOYSSR_19_1243	19:43023466-43533756	NA	NA
phQTL_19b^b–d^	BARCSOYSSR_19_1286-BARCSOYSSR_19_1532	19: 44370710-49060065	*Glyma.19G224300*	GM_19 (ss107929955)
				GM_19_A (BARCSOYSSR_19_1452)
				GM_19_B (OSU_SNP_Glyma19g41210)

^a^Chromosome and physical base pair (bp) position derived from version Wm82.a2.v1.

^b^phQTL reported in Wang et al. (2012b).

^c^phQTL reported in Stasko et al. (2016).

^d^phQTL reported in Wang et al. (2012a).

^e^phQTL reported in the present study.

NA, not applicable.

Methods to determine the co-localization of *trans*-eQTLs with phQTLs were adapted from Christie et al. (2017). Base pair positions of consolidated phQTLs and a window using the average linkage disequilibrium (LD) block size (242 kb) around the marker to which *trans*-eQTLs mapped were used to determine phQTL/trans-eQTL co-localization. Linkage disequilibrium blocks were determined using the Haploview four-gamete method (Barrett et al., 2005). To determine if the number of co-localized *trans*-eQTLs and phQTLs was significantly greater than expected, *trans*-eQTLs were permuted across available markers on the genetic map. A threshold (alpha = 0.05) was set based on 1,000 permutations.

### 2.8 Identification of putative master regulators

Methods for identifying master regulators for eQTL hotspots were adapted from Wang et al. (2017). Genes within an up- and downstream 242-kb window (average LD block size for this population) of the hotspot SNP that also had *cis*-eQTLs mapping were identified as initial candidate master regulators. If no genes within the window were associated with *cis*-eQTLs, genes with no mapping e-traits were considered. From these *cis*-eQTLs or positional candidates, putative master regulators were selected according to predicted functions of transcription factor (TF) or signaling molecules (SM).

## 3 Results

### 3.1 Genetic map reconstruction and quantitative disease resistance to *Phytophthora sojae*

While phQTLs for QDR have been previously mapped in the F_9:11_ RIL population derived from a cross between Conrad (resistant) and Sloan (susceptible) (Stasko et al., 2016), in order to ensure that the phQTLs were directly relevant to the expression data collected in this study (**Supplementary Figure S2**), we mapped phQTLs using data from only the subset of 92 RILs for which RNA-seq, phenotypic data, and previous genotypic data were available (Stasko et al., 2016). A genetic map was constructed consisting of 1,122 markers assembled in 28 linkage groups, with most of the 20 soybean chromosomes represented as a single linkage group and chromosomes 5, 6, 7, 11, 12, 13, 17, and 19 each represented by two linkage groups (**Supplementary Table S1**). A suggestive phQTL, significant at the chromosome but not genome-wide level, on chromosome 18 and nonsignificant associations with regional peaks but nonsignificant logarithm of the odds (LOD) scores on chromosomes 1, 16, and 19a correspond to phQTLs previously identified in the full RIL population (**Supplementary Figure S2**; Stasko et al., 2016). In comparison to previous studies, reduced significance is expected due to the smaller subset of RILs.

### 3.2 Genetic architecture of gene expression in mock and inoculated treatments

**Figure 1:**
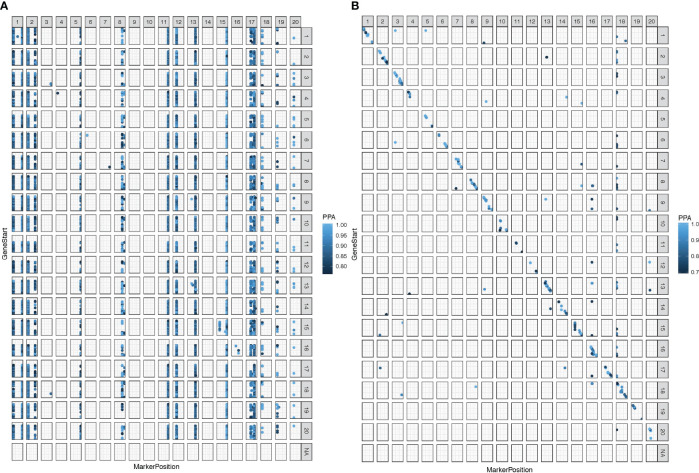
Expression quantitative trait loci (eQTL) were mapped in 92 Conrad × Sloan F_9:11_ RIL population at false discovery rate (FDR) of 10% with 100,000 iterations. **(A)** Significantly mapped inoculated treatment eQTL at a significance posterior probabilities of association (PPA) cutoff of 0.786 for the inoculated treatment. **(B)** Significantly mapped mock treatment eQTL at a significance PPA cutoff of 0.688. Base-pair positions are shown with zero at the bottom of the *y*-axis and right of the *x*-axis. Markers in a vertical formation indicate *trans*-eQTL, and markers in a diagonal formation indicate *cis*-eQTL. Chromosomes with no data contain nonclassified eQTL due to missing physical location data.

To identify the loci contributing to variation in gene expression, eQTLs were mapped under both mock and inoculated conditions. Gene expression levels were interpreted as quantitative traits (e-traits), and the locus or loci for each e-trait were mapped separately for inoculated and mock treatments. A total of 114,197 eQTLs were mapped from e-traits for the inoculated treatment from root samples collected 24 hai from the site of inoculation, representing transcripts from 35,781 unique genes associated with 74 unique loci (**Supplementary Table S2**). In contrast, the number of eQTLs mapped in the mock treatment was far lower but distributed across more loci, with 794 eQTLs representing 788 unique genes across 234 unique loci. The eQTLs identified in the inoculated treatment were distributed across most chromosomes, the exceptions being chromosomes 9 and 10 (**Figure 1A**), while eQTLs from the mock treatments were relatively evenly distributed across all chromosomes in the genome (**Figure 1B**). Interestingly, only two eQTLs were in common between mock and inoculated treatments, including the SNP, ss715580997, associated with e-trait *Glyma.02G107800* (putatively coding an uncharacterized protein), and the SNP OSU_SNP_Glyma19g41210, associated with e-trait, *Glyma.19G224300* (putatively coding a protein involved in regulation of root development and cell wall).

The eQTLs associated with e-traits of nearby genes are considered *cis*-eQTLs, while eQTLs altering the expression of physically distant genes are classified as *trans*-eQTLs (Hubner et al., 2005; Kliebenstein, 2009). In the inoculated treatment, the vast majority (111,568; ~98%) of the eQTLs were in the *trans* configuration (**Figure 1A**; **Supplementary Table S2**). In contrast, in the mock treatment, 83% (657) of the eQTLs were in the *cis* configuration (**Figure 1B**; **Supplementary Table S2**).

Key regulatory genes are expected to influence the expression of many genes. Therefore, eQTL hotspots, genomic regions enriched for eQTLs, are likely localized to these key regulatory genes (Kliebenstein, 2009; Tian et al., 2016). Thirty-six hotspots distributed across 12 chromosomes were identified for the inoculated treatment, with seven located on chromosome 17. Of the 36 hotspots in the inoculated treatment, there were two very large hotspots, GM_1 and GM_15, associated with 23,919 and 25,727 e-traits, respectively. For the mock treatment, there were three hotspots located on chromosomes 13, 16, and 18. The mock and inoculated eQTL hotspots did not overlap with each other (**Table 2**).

**Table 2:** Summary of inoculated and mock causal hotspots for number and regulation of expression quantitative trait loci (eQTLs) positively correlated to resistance towards *Phytophthora sojae*.

eQTL hotspot [associated co-expression module]^a^	Physical location^b^	Hotspot SNP^c^	Number of eQTLs
GM_1	1: 3033126	ss715578942^d^	23,919
GM_1_A	1: 3237203	ss715579001	110
GM_1_B	1: 49435179	Satt198	2,136
GM_2	2: 113491276	ss715580997	578
GM_2_A	2: 47857148	ss715583466	34
GM_2_B	2: 48192942	ss715583515^d^	16,909
GM_5 [Grey]	5: 1960856	ss715590397	423
GM_5_A	5: 2357871	ss715592433	24
GM_8	8: 3373388	ss715601478	44
GM_8_A	8: 15062941	ss715599654	367
GM_8_B	8: 15084953	ss715599658	119
GM_11	11: 3866567	ss715610573^d^	14,081
GM_12	12: 37942902	ss715612810	108
GM_12_A	12: 38033264	ss715612824	233
GM_12_B	12: 38161783	ss715612847	13,736
GM_12_C	12: 38314956	ss715612859	84
GM_13	13: 16051820	ss715616825^d^	6,889
GM_13_A [Honeydew, Darkred]	13: 14567149	ss715617113	20
GM_15	15: 4283809	ss715621908^d^	25,727
GM_15_A	15: 4334070	ss715621926	164
GM_15_B	15: 4425676	ss715621953	186
GM_15_C	15: 4541338	ss715622010	82
GM_17	17: 13225475	ss715626059^d^	6,966
GM_17_A	17: 17603614	ss715626313	59
GM_17_B	17: 24925330	ss715626528	29
GM_17_C	17: 28203907	ss715626623	93
GM_17_D [Honeydew, Darkred]	17: 31742522	ss715626724^d^	303
GM_17_E	17: 32295875	ss715626744	95
GM_17_F	17: 33008592	ss715626767	120
GM_18	18: 49185950	ss715631455^d^	91
GM_18_A	18: 49536028	ss715631507^d^	88
GM_19	19: 47232949	ss107929955	30
GM_19_A	19: 47528159	BARCSOYSSR_19_1452	34
GM_19_B	19: 47633059	OSU_SNP_Glyma19g41210	67
GM_20	20: 36332679	ss715637679	41
GM_20_A	20: 36720824	ss715637735	40
Mock GM_13	13: 30875555	ss715615049^d^	31
Mock GM_16	16: 34372952	ss715624691^d^	22
Mock GM_18	18: 57425465	ss715632465^d^	55

^a^Module listed in brackets indicates a significant association between the posterior probability of association (PPA) value and the respective module eigengene expression value at p-value <0.001.

^b^Chromosome and physical base pair (bp) position derived from version Wm82.a2.v1.

^c^Hotspots identified based on the SNP regulating the expression of >20 genes.

^d^Genes controlled by this SNP have significant gene ontology (GO) enrichment at a false discovery rate (FDR) of 5%.

### 3.3 Term enrichment and functional annotations of genes associated with each hotspot

Genes that are co-regulated may be associated with specific coordinated functions or resistance mechanisms. To identify a commonality of functions among e-traits mapping to each eQTL hotspot, GO term enrichment analysis was done. The e-traits associated with 10 of the 36 hotspots for the inoculated treatment were significantly enriched (*p*-value ≤ 0.05) for biological and/or cellular processes and molecular function terms. We were particularly interested in the enrichment of biological process GO terms, as enrichment of these terms may provide clues to the mechanisms of QDR. The biological process GO terms were significant for the e-traits associated with six of the 36 hotspots (**Supplementary Table S3**). A total of 16 and 31 biological processes were enriched among e-traits mapping to the large hotspots GM_1 (23,919 e-traits) and GM_15 (25,727 e-traits), respectively. Some of the most significantly enriched biological process terms were “intracellular transport,” “gene expression,” and “cellular processes” (**Supplementary Table S3**). Similar terms were also found for GM_2_B, GM_11, and GM13 as well as “intracellular signal transduction”. Four additional hotspots (GM_17_D, GM_17_F, GM_18, and GM_18_A) were not enriched for biological process GO terms but were enriched for cellular and molecular function GO terms. Overall, biological process GO term enrichment provided evidence of a functional relationship among e-traits within 10 hotspots and hinted at cell-to-cell signaling and protein modification roles but did not elucidate their involvement in a specific mechanism or pathway for QDR.

GO term enrichment for the e-traits associated with the three mock eQTL hotspots was also identified but revealed very general terms (**Supplementary Table S4**). While e-traits associated with each of the three mock eQTL hotspots were enriched for GO terms, only e-traits associated with GM_18_M were enriched for biological process terms. These included the terms “regulation of cellular process” and “biological regulation.” Any significant enrichment of GO terms indicates functional relationships among the genes at a hotspot; however, these general terms do little to inform the specific role of the e-traits at these hotspots.

The e-traits associated with each eQTL hotspot were also examined for enrichment of genes predicted to be involved in specific mechanisms of QDR in soybeans by *P. sojae*. The e-traits for six of 36 hotspots from the inoculated treatment (GM_1, GM_1_B, GM_2_B, GM_11, GM_13, and GM_15) were significantly enriched for genes in the plant–pathogen interaction pathway, and one hotspot (GM_11) was additionally significantly enriched for genes in the isoflavonoid pathway (**Table 3**). These findings suggest that these six hotspots are most likely regulating genes involved in PAMP-triggered immunity, defense-related gene induction, and/or programmed cell death, implicating leucine-rich repeat (LRR) encoding and other PAMP-triggering genes as likely candidate genes.

**Table 3:** Pathway enrichment of expression quantitative trait loci hotspots.

Hotspot	KEGG pathway^a^			No. genes in hotspot
	Plant-pathogen interactions	Isoflavonoid	Phenylpropanoid	
GM_1	175^***^	3	1	23,919
GM_1_B	16^*^	1	0	2,136
GM_2	2	0	0	578
GM_2_B	115^*^	2	0	16,909
GM_5	5	0	0	423
GM_8	1	0	0	44
GM_8_A	2	0	0	367
GM_8_B	1	0	0	119
GM_11	115^***^	5^**^	0	14,081
GM_12_A	1	0	0	233
GM_12_B	79	1	0	13,736
GM_12_C	1	0	0	84
GM_13	51^***^	2	0	6,889
GM_15	174^**^	4	1	25,727
GM_15_A	2	0	0	164
GM_15_B	2	0	0	186
GM_17_D	1	0	0	303
GM_17	40	2	1	6,966
GM_18	1	0	0	91
GM_18_A	1	0	0	88
Mock GM_18	1	0	0	55
**No. genes in pathway**	314	17	297	

^a^Gene list derived from the KEGG pathway database (accessed, February 2018; http://www.genome.jp/kegg/pathway.html); of the six pathways assessed, only those with genes represented by e-traits within hotspots are shown.

^*^p-value < 0.05, ^**^p-value < 0.001, ^***^p-value < 0.0001—levels of significance (Fisher’s exact test with Benjamini–Hochberg correction; Fisher, 1935; Benjamini and Hochberg, 1995).

We identified no enrichment for hypothesized resistance mechanisms for e-traits mapping to the three hotspots from the mock treatment nor for the remaining 30 of 36 hotspots from the inoculated treatment. While these findings may indicate a lack of concerted functional relationships among e-traits mapping to these hotspots, it may also be due to a lack of statistical power in the smaller hotspots or that the associated e-traits may be involved in unknown or untested mechanisms of QDR.

### 3.4 Weighted gene co-expression network analysis in the Conrad × Sloan RIL population

**Figure 2:**
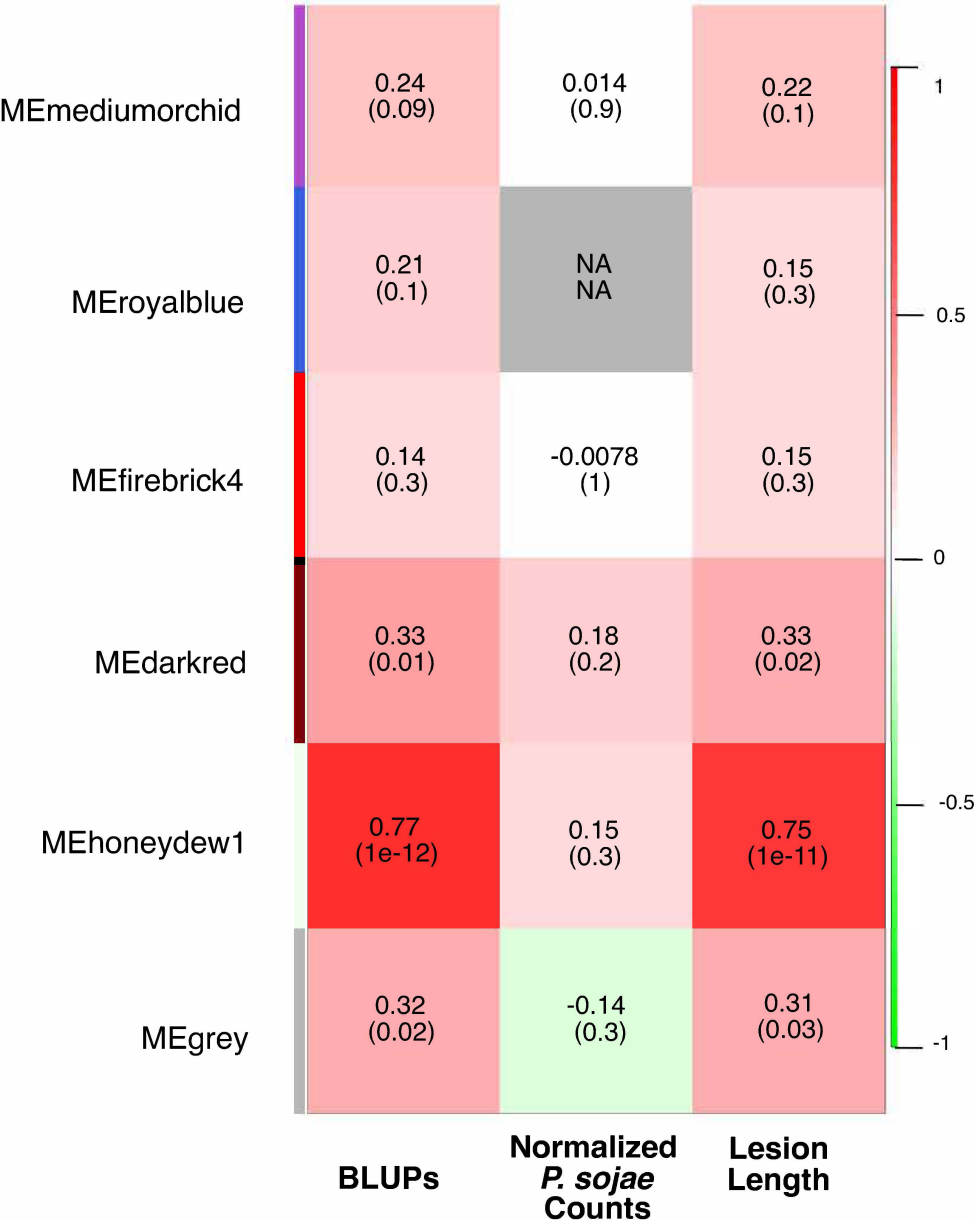
Consensus-weighted gene co-expression network analysis (WGCNA) for positively correlated modules. The best linear unbiased predictors (BLUPs) and lesion length values positively correlated to co-expression modules are in red, and those negatively correlated are in green. The first number represents the correlation coefficient, the number in parenthesis indicates the *p*-value. N/A indicates no consensus co-expression module was preserved.

To confirm and further characterize the transcriptional reprogramming that occurs following infection with *P. sojae* of this RIL population, we constructed expression networks, or weighted gene co-expression networks, determined by pairwise correlation of gene expression profiles (Langfelder and Horvath, 2007). This network analysis resulted in a total of six robustly defined modules (**Supplementary Figures S4**–**S6**). Module eigengene expression values from three modules, honeydew1, dark red, and grey, comprised of 20,976, 2,785, and 385 genes, respectively, had significant positive correlations with both BLUP values and lesion length for the PRR disease phenotype (**Figure 2**), indicating that as the expression of genes within these modules increased, susceptibility to PRR increased.

### 3.5 Term enrichment and functional annotations of co-expression modules

Following similar methods as to how we assessed the putative functions of eQTL hotspots, to ascertain the potential roles of the modules in susceptibility to PRR, GO term enrichment and functional annotations were assessed for genes within the honeydew1, dark red, and grey modules. While no significant GO enrichment was observed for the smaller dark red and grey modules, for the honeydew1 module, the most significantly enriched GO terms for biological processes included “translation,” “protein localization,” and “macromolecule localization” (**Supplementary Table S5**).

Genes within the co-expression modules were also evaluated for gene enrichment within the six known QDR pathways, for which we had previously evaluated e-traits in each hotspot for enrichment. The honeydew1 module was enriched for five of the six evaluated pathways: plant hormone signal transduction, phenylpropanoid biosynthesis, isoflavonoid biosynthesis, MAPK signaling, and plant-pathogen interaction pathways (**Table 4**). The dark red module was also significantly enriched for plant hormone signal transduction, MAPK signaling, and plant–pathogen interaction pathways. None of the six pathways were significantly enriched in the grey module, possibly due to the relatively smaller size of this module or indicating that there may be other pathways associated with resistance.

**Table 4:** Enrichment of genes within KEGG pathways for each co-expression module related to susceptibility.

KEGG pathway^a^		Plant hormone signal transduction	Phenylpropanoid biosynthesis	Isoflavonoid biosynthesis	MAPK signaling pathway	Plant-pathogen interaction	Total no. of genes in module
**Total No. of genes in the pathway**		587	297	17	281	314	–
**No. of pathway genes in the module**	**Honeydew1**	244	1^**^	8^***^	135^*^	160^***^	20,976
	**Darkred**	41^*^	0	0	27^**^	26^*^	2,785
	**Grey**	3	0	0	2	1	385

^a^Gene list derived from the KEGG pathway database (accessed, February 2018; http://www.genome.jp/kegg/pathway.html) and extracted from NCBI BioSystems (accessed, February 2018; https://www.ncbi.nlm.nih.gov/biosystems).

^*^p-value < 0.05, ^**^p-value < 0.001, ^***^p-value < 0.0001—levels of significance (Fisher’s exact test with Benjamini–Hochberg correction; Fisher, 1935; Benjamini and Hochberg, 1995).

### 3.6 Genetic architecture of co-expression modules

We integrated genetic markers associated with inoculated eQTL hotspots and co-expression modules (Zhang and Horvath, 2005) to further investigate the genetic loci influencing the three co-expression modules that were significantly correlated with PRR (honeydew1, dark red, and grey). The expression of each of the three module eigengenes was significantly correlated with the SNP(s) marking one or more of the eQTL hotspots. The correlation of an eQTL hotspot and a co-expression module to the same SNP suggests regulation of both by a common genetic mechanism (**Table 2**; **Supplementary Figures S7A–C**). The grey module was significantly correlated to the genetic marker for hotspot GM_5 (423 e-traits). The expression of both the honeydew1 and dark red module eigengenes was significantly correlated to SNPs from hotspots GM_13_A (20 e-traits) and GM_17_D (303 e-traits).

### 3.7 Co-localization of phQTLs and eQTLs

In order to understand the regulation of QDR by *P. sojae*, co-localizations of eQTLs or eQTL hotspots with phQTLs were identified. Among all of the 8 phQTLs mapped to unique locations in populations derived from Conrad × Sloan for *P. sojae* isolate 1.S.1.1 (Wang et al., 2010; Wang et al., 2012a; Stasko et al., 2016; **Table 1**), three phQTLs, which mapped to chromosomes 1, 18, and 19, co-localized with three hotspots and nine eQTLs (three inoculated *trans-* and six inoculated *cis-*eQTLs (**Table 1**). For both *cis*- and *trans*-eQTLs, this is significantly more co-localization than expected by random chance.

Co-localized with phQTL_1 were four *cis*-eQTLs for *Glyma.01G160600*, *Glyma.01G162600*, *Glyma.01G170600*, and *Glyma.01G171300* and two *trans*-eQTLs, both representing *Glyma.10G026500*, which had e-traits mapping independently to two markers within this phQTL region (**Table 1**). The e-traits of genes mapping in *cis* are towards genes annotated as a vacuolar iron transporter homolog, two-component response regulator ARR2, and an uncharacterized expressed sequence. The predicted protein of *Glyma.10G026500*, mapping to the two *trans*-eQTLs, was also uncharacterized. Interestingly, *just* outside (138 kb) of the average LD block size window (242 kb), which we used to consider co-localization between mapped *trans*-eQTL and phQTL, is the hotspot GM_1_B.

The e-traits for the paralogs *Glyma.18G270900* and *Glyma.08g249200* are both co-localized with phQTL_18b in *cis* and *trans*, respectively (**Table 1**). The gene sequences are present in syntenic blocks from the recent duplication within the soybean genome (Schmutz et al., 2010). Both genes are predicted to encode a malectin/receptor-like protein kinase.

Covering 4.7 Mb, phQTL_19b represented the largest genomic size of the phQTL we considered. As such, it co-localized with all three hotspots on chromosome 19 (GM_19, GM_19_A, and GM_19_B). However, these hotspots, all relatively small and possessing between 30 and 67 e-traits, were not enriched for any function or GO annotation, providing little evidence of a coordinated function. However, in addition to co-localization with the three hotspots, phQTL_19b also co-localized with both the mock and inoculated *cis*-eQTLs for the *Glyma.19G224300* e-traits.

### 3.8 Identification of master regulators controlling the expression of downstream suites of genes

Master regulators control the expression of a suite of downstream genes. To identify master regulators in QDR for *P. sojae*, we examined the key eQTL hotspots, which we defined as those that were significantly enriched for e-traits, correlated with modules, or co-localized with phQTL. From the 36 total eQTL hotspots, our primary and meta-analyses identified 16 key eQTL hotspots through the significant pathway or GO term enrichment of e-traits within a hotspot (GM_1, GM_1_B, GM_2_B, GM_11, GM_13, GM_15, GM_17, GM_17_D, GM_17_F, GM_18, GM_18_A), by the significant correlation of co-expression modules (GM_5, GM_13_A, GM_17_D), and/or the co-localization with a phQTL for resistance to *P. sojae* (GM_19, GM_19_A, GM_19_B). To better understand the regulation of these key hotspots, we identified their putative master regulators by integrating genetic position and putative gene function (methods adapted from Wang et al., 2017). We identified genes within the hotspot regions predicted to encode transcription factors or signaling molecules as putative master regulators (**Table 5**).

**Table 5:** Candidate master regulators of key hotspots.

eQTL hotspot^a^	Candidate master regulators^b^	Description^c^	TF/SM/*cis*-eQTL^d^
**GM_1** ^e,f^ **(23,919)**	*Glyma.01G017000*	3-Phosphoinositide-dependent protein kinase-1, putative	SM/*cis*-eQTL
	*Glyma.01G021000*	Auxin response factor 19	SM/*cis*-eQTL
	*Glyma.01G022000*	Methyl-CPG-binding domain protein 02; IPR011124 (zinc finger, CW-type)	TF/*cis*-eQTL
	*Glyma.01G002400*	Phospholipase A2 family protein	SM/*cis*-eQTL
**GM_1_B** ^f,^ **^i^ (2,136)**	*Glyma.01G156200*	Membrane transport protein, auxin efflux carrier	SM/*cis*-eQTL
	*Glyma.01G156600*	Thioredoxin reductase, pyridine nucleotide disulfide oxidoreductase	SM/*cis*-eQTL
	*Glyma.01G156700*	Hydroxymethylglutaryl-CoA reductase, mevalonate pathway 1	SM/*cis*-eQTL
**GM_2_B** ^e,f^ **^,j^ (16,909)**	*Glyma.02G306300*	WRKY DNA-binding domain, zinc-dependent activator protein	TF/*cis*-eQTL
	*Glyma.02G307300*	Aldo/keto reductase family, flavonoid biosynthesis-	SM/*cis-*eQTL
	*Glyma.02G307900* ^k^	FIMBRIN/PLASTIN, Ca^2+^-binding actin-bundling protein, EF-Hand protein superfamily	TF/*cis-*eQTL
	*Glyma.02G308800*	5′-AMP-activated protein kinase beta subunit, interaction domain, involved assembly of snf1 protein complex	SM/*cis-*eQTL
	*Glyma.02G309100*	Zinc finger, C3HC4 type (RING finger), E3 Ubiquitin protein ligase	TF/*cis-*eQTL
	*Glyma.02G310400*	Leucine-rich repeat receptor-like protein kinase, BR-signaling kinase 1	SM/*cis*-eQTL
**GM_5^h^ (423)**	*Glyma.05G021300*	Zinc-finger double-stranded RNA-binding, DNAJ homolog	TF/*cis-*eQTL
	*Glyma.05G021800*	Cytochrome P450, CYP2 subfamily, leucopelargonidin and leucocyanidin biosynthesis	SM/*cis-*eQTL
	*Glyma.05G021900*	Cytochrome P450, CYP2 subfamily, leucopelargonidin and leucocyanidin biosynthesis	SM/*cis-*eQTL
**GM_11** ^e,f,g^ **(14,801)**	*Glyma.11G051500*	Mn^2+^ and Fe^2+^ transporters of the NRAMP family, natural resistance-associated macrophage protein	SM/*cis*-eQTL
	*Glyma.11G052100*	Myb-like DNA-binding domain, TF, MYB superfamily	TF/*cis-*eQTL
	*Glyma.11G053100*	WRKY DNA-binding protein, transcription factor	TF/*cis*-eQTL
**GM_13** ^e,f^ **^,j^ (6,889)**	*Glyma.13G062400*	Reticulon	SM/*cis-*eQTL
	*Glyma.13G062700* ^k^	Glycosyl transferases group 1, glycogen biosynthesis (ADP-d-glucose)	SM/*cis-*eQTL
**GM_13_A^h^ (20)**	*Glyma.13G049000*	GDSL/SGNH-like acyl-esterase family found in Pmr5 and Cas1p	SM/*cis*-eQTL
**GM_15** ^e,f^ **(25,727)**	*Glyma.15G053600*	IPT/TIG domain, calmodulin binding transcription activator	TF/*cis-*eQTL
	*Glyma.15G053700*	Protein phosphatase 2C, serine/threonine protein phosphatase	SM/*cis-*eQTL
	*Glyma.15G054100*	Caspase domain, metacaspase is involved in the regulation of apoptosis	SM/*cis-*eQTL
	*Glyma.15G054500*	UDP-glucoronosyl and UDP-glucosyl transferase	SM/*cis-*eQTL
	*Glyma.15G054600*	Phosphate-induced protein 1 conserved region	SM/*cis-*eQTL
	*Glyma.15G054800*	RNA recognition motif (a.k.a. RRM, RBD, or RNP domain), splicing factor RNPS1, SR protein superfamily	SM/*cis-*eQTL
	*Glyma.15G055100*	EF-hand domain pair, calcium-binding protein	TF/*cis-*eQTL
	*Glyma.15G055200*	F-box domain	TF/*cis-*eQTL
	*Glyma.15G055500*	Amidohydrolase family, thymine degradation	SM/*cis-*eQTL
	*Glyma.15G056000*	Ring finger domain, anaphase-promoting complex	TF/*cis-*eQTL
**GM_17** ^e^ **(6,966)**	*Glyma.17G155500*	Kinesin motor domain	SM/*cis-*eQTL
**GM_17_D^h^ (303)**	*Glyma.17G200200*	C2H2 type zinc-finger	TF
	*Glyma.17G200500*	Transcription factor PCC	TF
**GM_17_F** ^e^ **(120)**	*Glyma.17G204300*	PHD finger proteins	TF
	*Glyma.17G204600*	Leucine-rich repeat receptor-like protein kinase, cytoplasmic	SM
**GM_18** ^e^ **(91)**	*Glyma.18G206400*	Leucine-rich repeat receptor-like protein kinase (serine/threonine)	SM
	*Glyma.18G207700*	Dirigent-like protein, disease resistance responsive	SM
	*Glym.18G208100*	Protein phosphatase 2C, serine/threonine protein phosphatase	SM
	*Glyma.18G208800*	WRKY DNA-binding domain	TF
	*Glyma.18G209400*	SWIM zinc finger	SM
**GM_18_A** ^e^ **(88)**	*Glyma.18G209400*	SWIM zinc finger	TF
	*Glyma.18G209500*	Wound-induced protein WI12	TF
**GM_19^i^ (30)**	*Glyma.19G217800*	WRKY DNA-binding	TF
	*Glyma.19G218800*	MYB-LIKE DNA-binding	TF
	*Glyma.19G219000*	MYB-LIKE DNA-binding	TF
	*Glyma.19G220000*	Zinc finger protein	SM
	*Glyma.19G220300*	Leucine-rich repeat receptor kinase (serine/threonine)	SM
**GM_19_A^i^ (34)**	*Glyma.19G221700*	WRKY DNA-binding domain (overlap with GM_19)	TF
**GM_19_B^i^ (67)**	*Glyma.19G222200*	MYB-LIKE DNA-binding (overlap with GM_19 and 19_A)	TF
	*Glyma.19G244200*	Two-component sensor histidine kinase—SNP in the gene (overlap with GM_19_A)	SM
	*Glyma.19G224600*	MYB-LIKE DNA binding (overlap with GM_19_A)	TF
	*Glyma.19G224700*	Basic helix-loop-helix/leucine zipper transcription factor (overlap with GM_19_A)	TF
	*Glyma.19G226000*	Interleukin-1 receptor-associated kinase (serine/threonine)	SM
	*Glyma.19G226900*	Zinc finger five domain-containing protein	SM

^a^Parenthetical number is the number of eQTL associated with each key hotspot. Key eQTL hotspots were those enriched QDR pathways based on the iBMQ analysis or overlap a phQTL of the Conrad × Sloan recombinant inbred population following inoculation with Phytophthora sojae.

^b^Candidate master regulators were identified as genes within a 242-kb (average linkage disequilibrium (LD) block size) upstream or downstream of the hotspot SNP, and genes with cis-eQTL mapping were identified as initial candidate master regulators. Genes were then selected as candidate master regulators if their putative function included transcription factors, signaling molecules, or known associations involved in the enriched quantitative disease resistance (QDR) pathways. Gene ID based on the Wms82.a2.v1 sequence (soybase.org).

^c^Description, PFAM, Panther, and pathway were retrieved from https://phytozome.jgi.doe.gov (accessed July 2023).

^d^Results from mapping genes to the KEGG Pathway database (accessed August 2023; http://www.genome.jp/kegg/pathway.html). Classification of the putative master regulator: putative signaling molecule (SM), putative transcription factor (TF), and cis-eQTL for this gene (cis-eQTL).

^e^Genes controlled by this SNP have significant gene ontology (GO) enrichment at a false discovery rate (FDR) of 5%.

^f^Significant pathway enrichment of the plant interaction pathway (adjusted p-value ≤ 0.05; Fisher’s exact test with Benjamini–Hochberg correction). Gene lists derived from the KEGG pathway database (accessed, February 2018; http://www.genome.jp/kegg/pathway.html).

^g^Significant pathway enrichment of the isoflavonoid pathway (p-value ≤ 0.05; Fisher’s exact test with Benjamini–Hochberg correction). Gene lists derived from the KEGG pathway database (accessed, February 2018; http://www.genome.jp/kegg/pathway.html).

^h^Significantly correlated with co-expression modules at a p-value <0.05 (Fisher’s exact test).

^i^Co-localizes with phenotypic quantitative trait loci (phQTL).

^j^Intragenic hotspot SNP.

^k^The hotspot SNP is located within this putative master regulator.

For nine of the 16 hotspots evaluated for master regulators (GM_1, GM_1_B, GM_2_B, GM_5, GM_11, GM_13, GM_13_A, GM_15, GM_17), we identified co-localized *cis*-eQTLs putatively encoding signal molecules and/or transcription factors (**Table 5**). To note, numerous hotspots were associated with multiple putative master regulators with genes for *cis*-eQTLs encoding both signaling molecules and transcription factors. For the four remaining eQTL hotspots (GM_17_D, MD_17_F, GM_18, and GM_18_A), while no genes within the LD window (242 kb) had *cis*-eQTLs, genes within the LD windows were putatively encoding transcription factors and/or signaling molecules and identified as candidate master regulators of these hotspots. In total, 15 genes putatively encoding transcription factors and 24 genes putatively encoding signaling molecules were identified as candidate master regulators for the 16 key eQTL hotspots (**Table 5**).

## 4 Discussion

Many phQTLs have been mapped through several generations in this Conrad × Sloan population (Wang et al., 2010; Wang et al., 2012a; Wang et al., 2012b; Stasko et al., 2016); however, identifying the mechanisms underpinning these phQTLs has, thus far, been unsuccessful. Studies using the resistant parent ‘Conrad’ have identified many putative mechanisms of quantitative resistance, demonstrating the complex nature of QDR and the potential that these mechanisms could be interacting (Vega-Sánchez et al., 2005; Thomas et al., 2007; Ranathunge et al., 2008; Zhou et al., 2009; Wang et al., 2012b). The application of a systems genomics approach in this study has allowed us to disentangle the complex genetic architecture of gene expression related to QDR for *P. sojae* using multiple approaches, including eQTL mapping, co-expression network analysis, and the co-localizations of phQTLs, eQTLs, and co-expression modules. The approaches taken in this study confirmed hypothesized mechanisms as well as provided evidence to suggest potential novel mechanisms of QDR for this pathosystem.

### 4.1 Inoculation with *P. sojae* causes transcriptional reprogramming that occurs in a *trans*-regulatory manner

Expression QTLs were successfully mapped in both the inoculated and mock treatments; however, there were 144-fold more eQTLs mapped for the inoculated treatment compared to the mock treatment. An average of two eQTLs per gene were mapped in the inoculated treatment, which is consistent with previous eQTL mapping studies (Schadt et al., 2003; Swanson-Wagner et al., 2009; Christie et al., 2017). Yet, in this study, only 794 eQTLs were identified for the mock, the majority of which were *cis*-eQTL. In stark contrast to the mock-inoculated treatment, nearly all eQTLs identified from the inoculated treatment in this study were *trans-*eQTLs (98%).

Gene expression has been shown in previous eQTL studies to be controlled by *trans*- or a combination of both *trans-* and *cis-*elements (West et al., 2007; Christie et al., 2017; Sun et al., 2017; Li et al., 2018). However, the number of eQTLs mapped, as well as the proportion of *trans*- vs. *cis*-eQTLs, has not followed a specific trend between species and populations (Keurentjes et al., 2007; West et al., 2007; Potokina et al., 2008; Swanson-Wagner et al., 2009; Hammond et al., 2011; Christie et al., 2017). For example, the number of eQTLs varied approximately ninefold between RIL populations in *Arabidopsis* (Keurentjes et al., 2007; West et al., 2007). While some studies have reported nearly equal ratios of *trans*- and *cis*-eQTLs detected in both *Arabidopsis* and barley (Keurentjes et al., 2007; Potokina et al., 2008), other studies have revealed a predominance of *trans*-regulation of eQTLs in *Arabidopsis* (86% and greater *trans*-eQTLs) (West et al., 2007; Soltis et al., 2020), barley (70% *trans*-eQTLs) (Druka et al., 2008), *Brassica rapa* (77% *trans*-eQTLs) (Hammond et al., 2011), and maize (up to 80% *trans*-eQTLs) (Swanson-Wagner et al., 2009; Christie et al., 2017). These varying results have been attributed to statistical power to detect *trans*-eQTLs, the size of the mapping population, the high polymorphism rate among genotypes in the study, and true biological differences between systems and their overall genetic architecture (Kliebenstein, 2009; Soltis et al., 2020). To date, only a few studies have been completed to address these questions of differing detection of eQTL types across plant species and populations (Franceschini et al., 2012; Saha and Battle, 2018).

The majority of plant-based eQTL studies to date have focused on natural genetic variation within breeding populations, different stages of maturation, or specific production or accumulation of compounds, and few have focused on mechanisms of disease resistance. Specifically in soybean, previous eQTL analyses identified predominantly *trans*-eQTLs for the genetic architecture of immature soybean seed (86.6%, *trans*-eQTLs) (Bolon et al., 2014) and dissection of isoflavonoid accumulation in soybean seed (60.6%, *trans*-eQTLs) (Wang et al., 2014).

The large number of *trans*-eQTLs mapped in this study were primarily associated with only eight eQTL hotspots, indicating massive transcriptional reprogramming resulting from the inoculation of soybean with *P. sojae.* This confirms several previous studies for quantitative resistance (Zhou et al., 2009; Soltis et al., 2020) and *Rps*-gene-related responses (Lin et al., 2014; Hale et al., 2023b). The eQTL hotspots identified in this study at 24 hai may represent key regulatory hubs and the control of signaling networks specifically in response to infection by *P. sojae.* More importantly, none of the 36 hotspots identified from the inoculated treatment overlap with the three hotspots identified from the mock treatment. This suggests that these hotspots in the mock represent either constitutive differences in regulation between Conrad and Sloan or a response specific to the mock treatment at the 24-hai time point. Of the few eQTL studies that have mapped transcriptional responses to disease, only a fraction of these studies compared eQTLs mapped in disease versus non-disease conditions in plant systems. Moscou et al. (2011), using microarrays to assay transcripts in barley following both inoculations with *Puccinia graminis* and mock inoculation, had findings that differed from this study, with similar numbers of eQTLs mapped in both mock and inoculated samples and the majority classified as *cis*. Here, the differences in the number of eQTL mapped between treatments, the lack of concordance of the hotspots between the mock and inoculated treatments, the correspondence with phQTLs, and the functional enrichment of genes within hotspots together suggest that the changes in transcription are due to infection by *P. sojae* through a coordinated transcriptional response of multiple plant defense mechanisms.

### 4.2 Major eQTL hotspots and co-expression networks elucidated potential QDR mechanisms, including signal integration and defense action via cell wall strengthening

Expression QTL hotspots are a single polymorphism associated with the expression of numerous genes (Neto et al., 2012), and the genetic regions may harbor important regulatory genes. In this study, the four largest hotspots, mapping to chromosomes 1, 2, 11, and 15, accounting for more than 80,000 eQTLs (>70%) from the inoculated treatment, were each enriched for genes within the plant–pathogen interaction (PPI) pathways. Specifically, PPI pathway genes found within the hotspots were predicted to function throughout the pattern-triggered immunity (PTI) pathway. The separation of the PTI and ETI pathways within the context of plant resistance to oomycetes has recently come into question in favor of a three-layer plant immune system (consisting of the recognition, signal integration, and defense-action layers) describing both PTI and ETI for plant-pathogenic oomycete infection (Wang et al., 2019; Naveed et al., 2020). The signal-integration layer represents a complex network of pathway cascades including phosphorylation, ubiquitination, relocation, degradation, stabilization of proteins, transcriptional regulation, and chemical signaling (Wang et al., 2019). The QDR pathway enrichment within these major hotspots, along with the significantly enriched GO terms related to cell-to-cell signaling and protein modification, support the involvement of these hotspots in the signal-integration layer of defense. This aligns with previously implicated defense mechanisms in plant–oomycete interactions and specifically within the *P. sojae*-soybean pathosystem (Wang et al., 2012b; Wang et al., 2019).

These four eQTL hotspots represent genetic variation for transcriptional reprogramming resulting from inoculation with *P. sojae*, a phenomenon that has been previously reported (Zhou et al., 2009; Wang et al., 2010). Yet, these hotspots do not localize to the regions of any phQTLs identified in this study or previous studies. Samad-Zamini et al. (2017) had similar findings, where none of the hotspots co-localized with phQTLs during a time-course assay of *Fusarium graminearum* (FHB) infection of wheat (*Triticum aestivum* L.). This lack of co-localization may be due to residual genetic variation of e-traits not significantly attributed to phQTL; this variation may involve complex genetic interactions, including epistasis (Li, 2019).

In this study, we also identified a total of 24,146 genes within three co-expression modules that were significantly correlated to the PRR disease resistance response. The SNPs corresponding to two hotspots, GM_13_A and GM_17_D, were also both significantly correlated to the co-expression modules dark red and honeydew1. GM_17_D was enriched for GO terms including “hydrolase activity” and “cell wall structure,” functions that align with the third layer of resistance, defense-action (Wang et al., 2019). Genes involved in the modification of the cell wall and hydrolase activity have been shown to be involved in plant defense responses (Smith et al., 1988; Smith et al., 1990; Walton, 1994; Minic, 2008). Hydrolase expression has been previously associated with quantitative resistance to *P. sojae* with specific hydrolases suppressed at 48 hai in the resistant parent Conrad and suppressed at 72 hai in the susceptible parent Sloan (Wang et al., 2012b). The GM_17_D hotspot may be involved in the coordinated regulation of these two modules, and importantly, these co-expressed genes may play a role in limiting pathogen penetration into the cell wall in response to pathogen presence. The dark red and honeydew1 co-expression modules, along with the third co-expression module, grey, were also each enriched for genes involved in the plant hormone signal transduction, MAPK signaling pathway, and PPI pathways, providing evidence for coordinated regulation of expression among each of these defense mechanisms.

The honeydew1 module was further described by its additional enrichment of genes from the phenylpropanoid biosynthesis and isoflavonoid pathways. The role of phenylpropanoid and isoflavonoid in the *R*-gene-mediated response has been well studied (Graham et al., 2007), including the recent identification of a transcription factor that modulates this response (Jahan et al., 2020). However, the specific role of phenylpropanoid and isoflavonoid pathways in QDR has been more elusive. Gene expression in the honeydew1 module is correlated with increased susceptibility, supporting recent evidence in the cross-talk that occurs between the pathways for *R*-gene-mediated and QDR. Previous studies showed both SA and JA increasing at inoculated roots, with JA further increased in later time points after inoculation (Stasko et al., 2020; Karhoff et al., 2022). This 24-hai time point could be a critical time as the pathogen switches from hemibiotrophy to the necrotrophic phase (Moy et al., 2004). Several genes in the phenylpropanoid pathway have been identified as playing a role in resistance to *P. sojae* in soybeans. For example, soybean cinnamate 4-hydrolase (GmC4H1; first hydroxylation step of the phenylpropanoid pathway) was induced at 24 hai in the resistant parent Conrad, and greater colonization of *P. sojae* was measured in *GmC4H1*-silenced plants (Yan et al., 2019). Recently, in the wheat-*Fusarium graminearum* system, it was reported that wheat genotypes with greater levels of resistance had a constitutive expression of genes for plant cell wall biogenesis and terpene biosynthesis (Buerstmayr et al., 2021).

### 4.3 Genetic regulation of trans-eQTLs in response to the pathogen is predicted to occur through TF and signaling molecules involved in PPI, plant hormone signal transduction, and novel mechanisms of resistance

Numerous studies have proposed that *cis*-acting mechanisms (i.e., transcription factors) can affect the expression of e-traits at *trans*-eQTL hotspots (Albert and Kruglyak, 2015; Wang et al., 2017). Thus, *cis*-eQTLs located near regulatory genes have the potential to be master regulators for these e-traits in a given hotspot (Bryois et al., 2014; Albert and Kruglyak, 2015; Yao et al., 2015). We identified candidate master regulators for the key hotspots that had significant GO or pathway enrichment, were correlated to a co-expression module, and/or were co-localized with phQTL. Of these, four putative master regulators were predicted to function in the PPI pathway as LRR-RLKs, or EF-hand motif proteins.

LRR-RLKs were predicted to be encoded by candidate master regulators for GM_2_B, GM_17F, GM_18, and GM_19. These LRR-RLKs are crucial for plant function and adaptation in numerous processes such as growth and development, as well as responses to abiotic and biotic stresses (Chinchilla et al., 2009; De Smet et al., 2009). Among their numerous functions, LRR-RLKs are known to function in all three layers of defense through the perception of microbe-associated molecular patterns resulting in a basal defense (e.g., *FLS2*), defense signaling (e.g., *SIF2*), and defense response (e.g., *PEPR2*) (Gómez-Gómez and Boller, 2000; Becraft, 2002; Shiu et al., 2004; Zipfel et al., 2006; Yamaguchi et al., 2010; Kemmerling et al., 2011; Soyars et al., 2016; Hohmann et al., 2017; Zipfel and Oldroyd, 2017; Yuan et al., 2018; Wang et al., 2019). LRR-RLKs have been identified as candidates for resistance to *Phytophthora* spp. in numerous studies including this *P. sojae*-soybean system (Schneider et al., 2016; Stasko et al., 2016; Rolling et al., 2020).

Two EF-hand motif proteins were each identified as candidate master regulators associated with the PPI pathway for hotspots GM_2_B and GM_15, respectively, with GM_2_B being a hotspot enriched for genes within the PPI pathway. Approximately 250 EF-hand motifs have been identified in plants and are involved with Ca^2+^, which acts as a messenger that regulates responses to external stimuli, development, and hormones, including plant defense and stress response (Poovaiah and Reddy, 1993; Trewavas and Mahlo, 1998; Reddy and Reddy, 2001; Zielinski, 1998). The majority of Ca^2+^ sensors in soybeans possess the EF-hand motif and have at least one or more hormone- or stress-response-related *cis*-elements in their promoter region (Zeng et al., 2017). These hormone- or stress-response-related elements have been characterized by functioning in the regulation of abscisic acid (ABA) signaling, auxin response, ethylene response, and phosphate starvation response. Of these signaling and responses potentially regulated by EF-hand motif encoding genes, ABA has been shown to be a negative regulator of *R*-gene-mediated resistance (Ward et al., 1989; MacDonald and Cahill, 1999), and auxin has been reported to enhance plant susceptibility to *P. sojae* in soybean (Stasko et al., 2020) and other pathogens (Wang et al., 2007; Domingo et al., 2009; Kidd et al., 2011). Auxin transporters and auxin-induced proteins have been upregulated in susceptible parents in the *P. sojae*-soybean pathosystem (Wang et al., 2012b). Auxin transport transcripts of *GmPIN* were higher in expression in the resistant Conrad following inoculation with *P. sojae* compared to mock, whereas in the susceptible, fewer *GmPIN* changed in expression levels (Stasko et al., 2020). Additionally, ethylene-responsive genes have also been known to induce resistance in the *P. sojae*-soybean pathosystem (Sugano et al., 2013; Zhao et al., 2017), as well as play a role in the regulation of pathogenesis-related gene expression (Lorenzo et al., 2003; Pieterse et al., 2009; Rehman and Mahmood, 2015). Taken together, the EF-hand motif-encoding genes are excellent candidate master regulators for GM_2_B and GM_15.

In addition to these candidate master regulators within the PPI pathway and those that overlap phQTLs, several MYB-TFs were identified as candidate master regulators for GM_11 and GM_19, GM_19_A, and GM_19_B. MYB transcription factors are one of the six major TF families functioning in plant defense (Ng et al., 2018), responding to both abiotic and biotic stresses, and functioning in primary and secondary metabolism (Stracke, 2001; Ambawat et al., 2013), including the regulation of the phenylpropanoid pathway (Liu et al., 2015). Additionally, GmMYB29A2 is essential for the *R*-gene response to *P. sojae* in soybeans, regulating the accumulation of glyceollin in Williams 82 (Jahan et al., 2020), and *MYB* transcripts were also detected by capture-seq from a transcriptome data set of the *R*-gene response in Williams 82 (Hale et al., 2023b). They are known to act as a positive regulator of hypersensitive response in PCD in response to fungal and bacterial pathogens (Vailleau et al., 2002). Thus, these may be putative master regulators involved with the positive regulation of PCD in the soybean-*P. sojae* pathosystem.

GM_17_D did not have any *cis*-eQTLs mapping to this hotspot. However, significant differential expression is not a requirement for a master regulator. For example, *hunchback* (*hb*), encoding a ZN-finger TF in *Drosophila melanogaster*, was identified as a candidate master regulator for mitigation of lead exposure, located near a *trans*-eQTL hotspot, yet the candidate itself had no e-traits mapped (Qu et al., 2018). Here, we note that the zinc finger TF (*Glyma.17G200200*) is a candidate master regulator of GM_17_D because it is not only physically located near the hotspot but is also within the honeydew1 co-expression network that is correlated with PRR disease.

In addition to the candidate master regulators functioning in known or hypothesized pathways for QDR, we also identified candidate master regulators that putatively influence novel pathways for QDR. These novel pathways for QDR in *P. sojae* included secondary metabolite biosynthesis, RNA transport, thioredoxin metabolism (GM_1_B), lysine degradation (Glyma_2_B), reticulon (Lee et al., 2011), starch and sucrose metabolism (GM_13), thymine degradation (GM_15), and a number of serine/threonine protein kinases and phosphatases that impact other metabolic pathways. These candidate master regulators of novel QDR pathways include TFs and signaling molecules that potentially regulate the expression of downstream genes related to hotspots. Further studies will be needed to determine if and how these pathways are playing a role in the *P. sojae*-soybean pathosystem.

### 4.4 Co-localization of phQTLs with eQTL points to causal candidate genes for QDR

To identify gene expression variation that may be causal to PRR disease resistance, we focused on those eQTLs that co-localized with phQTLs, indicating a strong link between transcriptional phenotype and the genes underpinning the disease resistance phenotype. Specifically, co-localized *cis*-eQTLs, the genes regulating co-localized *trans*-eQTLs, or *trans*-eQTL hotspots may be causal for resistance to PRR.

While none of the four *cis*-eQTLs co-localized with phQTL_1 have obvious functions in quantitative disease resistance, the hotspot GM_1_B, which neighbors phQTL_1, is enriched for genes functioning in PPI pathways, making regulators of this hotspot viable causal genes for phQTL_1. Three *cis*-eQTLs were identified as candidate master regulators for this hotspot: *Glyma.01G156600*, *Glyma.01G156200*, and *Glyma.01G156700*. These genes are predicted to encode a thioredoxin reductase, a membrane transport protein, and a hydroxymethylglutaryl-CoA reductase, respectively. The predicted thioredoxin reductase (*Glyma.01G156600*) is of interest given the role of thioredoxin in disease resistance and potentially QDR to *P. sojae*, with thioredoxin-encoding genes identified as candidate genes for several quantitative disease resistance loci (QDRL) towards *P. sojae* (Huang et al., 2016; Stasko et al., 2016). Additionally, a thioredoxin-encoding gene has been shown to be the causal gene for resistance at the *Scmv1* phQTL for sugarcane mosaic virus in maize (Liu et al., 2017). The predicted membrane transport protein encoded by *Glyma.01G156200* is an auxin efflux carrier. Auxin has been previously described in numerous studies as being involved in susceptibility to plant pathogens (Wang et al., 2007; Domingo et al., 2009; Kidd et al., 2011; Pieterse et al., 2012). Finally, hydroxymethylglutaryl-CoA reductase, predicted to be encoded by *Glyma.01G156700*, is involved in terpenoid and secondary metabolite biosynthesis (Antolin-Llovera et al., 2011). Thus, these potential master regulators for the Gm_1_B hotspot may represent the causal genetic variation for phQTL_1.

The phQTL_18b was co-localized with two eQTLs controlling the expression of *Glyma.18G270900* and *Glyma.08g249200* in *cis* and *trans*, respectively, each putatively encoding a malectin/receptor-like protein kinase. In *Arabidopsis*, the homolog of these genes, *FERONIA*, has been experimentally shown to have multiple functions, including as a modulator of ethylene response (Deslauriers and Larsen, 2010) and in reactive oxygen species (ROS)-mediated root hair development (Duan et al., 2010). ROS is a well-known mediator of stress-induced responses and functions in growth and development (Werner, 2004; Swanson and Gilroy, 2010; Torres, 2010). *FERONIA* also functions to inhibit jasmonic acid (JA) signaling through phosphorylation of the transcription factor MYC2 in Arabidopsis (Guo et al., 2018). In soybean, a role for JA was proposed in the later stages of infection by *P. sojae* (Stasko et al., 2020). The JA pathway was suppressed in incompatible *R*-gene reactions to *P. sojae* (Lin et al., 2014), and JA accumulation significantly increased in *P. sojae*-inoculated susceptible lines in contrast to the mock-inoculated and to lines with quantitative resistance alleles (Karhoff et al., 2022).

In addition to co-localization with the three hotspots (GM_19, GM_19_A, and GM_19_B), phQTL_19b co-localized with both the mock and inoculated *cis*-eQTLs for the *Glyma.19G224300* e-traits. This inoculated *cis*-eQTL is also part of the GM_19_B eQTL hotspot. The e-traits for *Glyma.19G224300* represented one of only two pairs of e-QTLs that were found under both mock and inoculated conditions, indicating possible constitutive control of both GM_19_B and of phQTL_19b. *Glyma.19G224300* is predicted to encode a germin-like protein (GLP). Among their functions, GLPs can be involved in response to abiotic stress (Barman and Banerjee, 2015). In *Arabidopsis*, upregulation of the *Glyma.19G224300* homolog *AT1G09560* results in reduced primary root and enhanced lateral root growth (Ham et al., 2012). *Glyma.19G224300* may function in root architecture, providing constitutive quantitative resistance to *P. sojae*, with the differences in disease resulting from expression changes mapping to GM_19_B.

## 5 Concluding remarks

This vast transcriptional reprogramming due to pathogen infection compared to the nondisease state had not been previously explored through eQTL methodology using RNA-sequencing data in this host–pathogen system. Ultimately, this study identified gene co-expression modules associated with resistance and susceptibility to *P. sojae* in this RIL population. Clearly, the transcriptional response to this pathogen is complex, as there were more than 100-fold greater number of eQTLs in the inoculated compared to the mock treatment, as well as a predominance of *trans*-eQTLs in the inoculated over the mock treatment. Further evidence supporting cell wall structure, auxin response, jasmonic acid signaling, and PPI receptor and signaling genes as mechanisms of resistance are provided, as well as several new potential mechanisms for regulating resistance as well as potential susceptibility factors. Further confirmation of the candidate genes regulating *trans*-eQTLs and/or acting as the causal variation of phQTLs will need to be explored through functional studies. The development of this large dataset and analyses through co-expression networks, eQTLs, and phQTLs have the potential to be expanded to elucidate more biologically relevant information on *P. sojae* infection as well as constitutive differences between two cultivars.

## Data availability statement

The datasets presented in this study can be found in online repositories. The names of the repository/repositories and accession number(s) can be found below: https://www.ncbi.nlm.nih.gov/, PRJNA478334.

## Author contributions

CM: Data curation, Formal analysis, Investigation, Methodology, Visualization, Writing – original draft, Writing – review & editing. SW: Formal analysis, Methodology, Supervision, Writing – review & editing. SK: Formal analysis, Investigation, Writing – original draft, Writing – review & editing. BC: Conceptualization, Funding acquisition, Investigation, Writing – review & editing. LM: Conceptualization, Methodology, Supervision, Writing – original draft, Writing – review & editing. AD: Methodology, Resources, Supervision, Writing – original draft, Writing – review & editing.

## Supplementary material

Methods M1 Constructing 20 networks to use as reference networks for input into module consensus analysis R script.

Methods M2 Creating consensus network from two selected reference modules R script.

Methods M3 Correlation of hotspots to co-expression modules R script.

## References

[B1] Abbasi P. A. Graham M. Y. Graham T. L. (2001). Effects of soybean genotype on the glyceollin elicitation competency of cotyledon tissues to *Phytophthora sojae* glucan elicitors. Physiol. Mol. Plant Pathol. 59, 95–105. doi: 10.1006/pmpp.2001.0342

[B2] Abeysekara N. S. Matthiesen R. L. Cianzio S. R. Bhattacharyya M. K. Robertson A. E. (2016). Novel sources of partial resistance against *Phytophthora sojae* in soybean PI 399036. Crop Sci. 56, 2322–2335. doi: 10.2135/cropsci2015.09.0578

[B3] Acharjee A. Chibon P. Kloosterman B. America T. Renaut J. Maliepaard C.. (2018). Genetical genomics of quality related traits in potato tubers using proteomics. BMC Plant Biol. 18, 20. doi: 10.1186/s12870-018-1229-1 29361908 PMC5781343

[B4] Albert F. W. Kruglyak L. (2015). The role of regulatory variation in complex traits and disease. Nat. Rev. Genet. 16, 197–212. doi: 10.1038/nrg3891 25707927

[B5] Ambawat S. Sharma P. Yadav N. R. Yadav R. C. (2013). MYB transcription factor genes as regulators for plant responses: an overview. Physiol. Mol. Biol. Plants 19, 307–321. doi: 10.1007/s12298-013-0179-1 24431500 PMC3715649

[B6] Andrews S. (2010). FastQC: A quality control tool for high throughput sequence data. Available at: http://www.bioinformatics.babraham.ac.uk/projects/fastqc/.

[B7] Antolin-Llovera M. Leivar P. Arro M. Ferrer A. Boronat A. Campos N. (2011). Modulation of plant HMG-CoA reductase by protein phosphates 2A positive and negative control at a key node of metabolism. Plant Signal Behav. 8, 1127–1131. doi: 10.4161/psb.6.8.16363 PMC3260709 21701259

[B8] Arahana V. S. Graef G. L. Specht J. E. Steadman. J. R. Eskridge K. M. (2001). Identification of QTLs for resistance to *Sclerotinia sclerotiorum* in soybean. Crop. Sci. 41, 180–188. doi: 10.2135/cropsci2001.411180x

[B9] Ausubel F. M. (2005). Are innate immune signaling pathways in plants and animals conserved? Nat. Immunol. 6, 973–979. doi: 10.1038/ni1253 16177805

[B10] Barman A. R. Banerjee J. (2015). Versatility of germin-like proteins in their sequences, expressions, and functions. Funct. Integ. Genomics 15, 533–548. doi: 10.1007/s10142-015-0454-z 26174051

[B11] Barrett J. C. Fry B. Maller J. Daly M. J. (2005). Haploview: Analysis and visualization of LD and haplotype maps. Bioinformatics 21, 263–265. doi: 10.1093/bioinformatics/bth457 15297300

[B12] Bates D. Maechler M. Bolker B. Walker S. (2014). Fitting linear mixed-effects models using lme4. J. Stat. Software 67, 1–48. doi: 10.48550/arXiv.1406.5823

[B13] Bayless A. M. Zapotocony R. W. Grunwald D. J. Amundson K. K. Diers B. W. Bent A. F. (2018). An atypical N-ethylmaleimide sensitive factor enables the viability of nematode-resistant *Rhg1* soybeans. Proc. Natl. Acad. Sci. U.S.A. 115, E4512–E4521. doi: 10.1073/pnas.1717070115 29695628 PMC5948960

[B14] Becraft P. W. (2002). Receptor kinase signaling in plant development. Annu. Rev. Cell Dev. Biol. 18, 163–192. doi: 10.1146/annurev.cellbio.18.012502.083431 12142267

[B15] Benjamini Y. Hochberg Y. (1995). Controlling the false discovery rate: A practical and powerful approach to multiple testing. J. R. Stat. Soc.: Ser. B (Methodological) 57, 289–300. doi: 10.1111/j.2517-6161.1995.tb02031.x

[B16] Boller T. Felix G. (2009). A renaissance of elicitors: perception of microbe-associated molecular patterns and danger signals by pattern-recognition receptors. Annu. Rev. Plant Biol. 60, 379–406. doi: 10.1146/annurev.arplant.57.032905.105346 19400727

[B17] Bolon Y. T. Hyten D. L. Orf J. H. Vance C. P. Muehlbauer G. J. (2014). eQTL Networks reveal complex genetic architecture in the immature soybean seed. Plant Genome 7, 1–14. doi: 10.3835/plantgenome2013.08.0027

[B18] Broman K. W. Wu H. Sen Ś. Churchill G. A. (2003). R/qtl: QTL mapping in experimental crosses. Bioinformatics 19, 889–890. doi: 10.1093/bioinformatics/btg112 12724300

[B19] Bryois J. Buil A. Evans D. M. Kemp J. P. Montgomery S. B. Conrad D. F.. (2014). Cis and trans effects of human genomic variants on gene expression. PLoS Genet. 10, e1004461. doi: 10.1371/journal.pgen.1004461 25010687 PMC4091791

[B20] Buerstmayr M. Wagner C. Nosenko T. Omony J. Steiner B. Nussbaumer T.. (2021). Fusarium head blight resistance in European winter wheat: insights from genome-wide transcriptome analysis. BMC Genomics 22, 1–17. doi: 10.1186/s12864-021-07800-1 34167474 PMC8228913

[B21] Burnham K. D. Dorrance A. E. VanToai T. T. St. Martin S. K. (2003). Quantitative trait loci for partial resistance to *Phytophthora sojae* in soybean. Crop Sci. 43, 1610–1617. doi: 10.2135/cropsci2003.1610

[B22] Bushnell B. (2014). BBMap: A fast, accurate, splice-aware aligner (Berkeley, CA: Ernest Orlando Lawrence Berkeley National Laboratory). Available at: https://www.osti.gov/servlets/purl/1241166.

[B23] Chen X. Hackett C. A. Niks R. E. Gedley P. Booth C. Druka A.. (2010). An eQTL analysis of partial resistance to *Puccinia hordei* in barley. PLoS One 5, e8598. doi: 10.1371/journal.pone.0008598 20066049 PMC2798965

[B24] Cheng Q. Li N. Dong L. Zhang D. Fan S. Jiang L.. (2015). Overexpression of soybean isoflavone reductase (*GmIFR*) enhances resistance to *Phytophthora sojae* in soybean. Front. Plant Sci. 6. doi: 10.3389/fpls.2015.01024 PMC4655237 26635848

[B25] Chinchilla D. Shan L. He P. de Vries S. Kemmerling B. (2009). One for all: The receptor-associated kinase BAK1. Trends Plant Sci. 14, 535–541. doi: 10.1016/j.tplants.2009.08.002 19748302 PMC4391746

[B26] Chisholm S. T. Coaker G. Day B. Staskawicz B. J. (2006). Host-microbe interactions: shaping the evolution of the plant immune response. Cell 124, 803–814. doi: 10.1016/j.cell.2006.02.008 16497589

[B27] Christie N. Myburg A. A. Joubert F. Murrasy S. L. Carstens M. Lin Y. C.. (2017). Systems genetics reveals a transcriptional network associated with susceptibility in the maize-grey leaf spot pathosystem. Plant J. 89, 746–763. doi: 10.1111/tpj.13419 27862526

[B28] Cook D. E. Bayless A. M. Wang K. Guo X. Song Q. Jiang J.. (2014). Distinct copy number, coding sequence, and locus methylation patterns underlie *Rhg1*-mediated soybean resistance to soybean cyst nematode. Plant Physiol. 165, 630–647. doi: 10.1104/pp.114.235952 24733883 PMC4044848

[B29] Cook D. E. Lee T. G. Guo X. Meltio S. Wang K. Bayless A. M.. (2012). Copy number variation of multiple genes at *Rhg1* mediates nematode resistance in soybean. Science 338, 1206–1209. doi: 10.1126/science.1228746 23065905

[B30] Corwin J. A. Copeland D. Feusier J. Subedy A. Eshbaugh R. Palmer C.. (2016). The quantitative basis of the *Arabidopsis* innate immune system to endemic pathogens depends on pathogen genetics. PLoS Genet. 12, e1005789. doi: 10.1371/journal.pgen.1005789 26866607 PMC4750985

[B31] Corwin J. A. Kliebenstein D. J. (2017). Quantitative resistance: More than just perception of a pathogen. Plant Cell 29, 655–665. doi: 10.1105/tpc.16.00915 28302676 PMC5435431

[B32] Cui H. Tsuda K. Parker J. E. (2015). Effector-triggered immunity: from pathogen perception to robust defense. Annu. Rev. Plant Biol. 66, 487–511. doi: 10.1146/annurev-arplant-050213-040012 25494461

[B33] Dastmalchi M. Chapman P. Yu J. Austin R. S. Dhaubhadel S. (2017). Transcriptomic evidence for the control of soybean root isoflavonoid content by regulation of overlapping phenylpropanoid pathways. BMC Genomics 18, 1–15. doi: 10.1186/s12864-016-3463-y 28077078 PMC5225596

[B34] de Ronne M. Santhanam P. Cinget B. Labbé C. Lebreton A. Ye H.. (2022). Mapping of partial resistance to *Phytophthora sojae* in soybean PIs using whole-genome sequencing reveals a major QTL. Plant Genome 15, e20184. doi: 10.1002/tpg2.20184 34964282 PMC12807403

[B35] Deslauriers S. D. Larsen P. B. (2010). FERONIA is a key modulator of brassinosteroid and ethylene responsiveness in *Arabidopsis* hypocotyls. Mol. Plant 3, 626–640. doi: 10.1093/mp/ssq015 20400488

[B36] De Smet I. Voss U. Jürgens G. Beeckman T. (2009). Receptor-like kinases shape the plant. Nat. Cell Biol. 11, 1166–1173. doi: 10.1038/ncb1009-1166 19794500

[B37] Dodds P. N. Rathjen J. P. (2010). Plant immunity: towards an integrated view of plant-pathogen interactions. Nat. Rev. Genet. 11, 539–548. doi: 10.1038/nrg2812 20585331

[B38] Domingo C. Andrés F. Tharreau D. Iglesias D. J. Talón M. (2009). Constitutive expression of OsGH3.1 reduces auxin content and enhances defense response and resistance to a fungal pathogen in rice. Mol. Plant Microbe Interact. 22, 201–210. doi: 10.1094/MPMI-22-2-0201 19132872

[B39] Dorrance A. E. Robertson A. E. Cianzo S. Giesler L. J. Grau C. R. Draper M. A.. (2009). Integrated management strategies for *Phytophthora sojae* combining host resistance and seed treatments. Plant Dis. 93, 875–882. doi: 10.1094/PDIS-93-9-0875 30754536

[B40] Druka A. Potokina E. Luo Z. Bonar N. Druka I. Zhang L.. (2008). Exploiting regulatory variation to identify genes underlying quantitative resistance to the wheat stem rust pathogen *Puccinia graminis* f. sp. *tritici* in barley. Theor. Appl. Genet. 117, 261–272. doi: 10.1007/s00122-008-0771-x 18542913

[B41] Druka A. Potokina E. Luo Z. Jiang N. Chen X. Kearsey M.. (2010). Expression quantitative trait loci analysis in plants. J. Plant Biotechnol. 8, 10–27. doi: 10.1111/j.1467-7652.2009.00460.x 20055957

[B42] Duan Q. Kita D. Li C. Cheung A. Y. Wu H. M. (2010). FERONIA receptor-like kinase regulates RHO GTPase signaling of root hair development. Proc. Natl. Acad. Sci. 107, 17821–17826. doi: 10.1073/pnas.1005366107 20876100 PMC2955125

[B43] Feltus F. A. (2014). Systems genetics: A paradigm to improve discovery of candidate genes and mechanisms underlying complex traits. Plant Sci. 223, 45–48. doi: 10.1016/j.plantsci.2014.03.003 24767114

[B44] Fisher R. A. (1935). The logic of inductive inference. J. R. Stat. Soc. 98, 39–82. doi: 10.2307/2342435

[B45] Franceschini A. Szklarczyk D. Frankild S. Kuhn M. Simonovic M. Roth A.. (2012). STRING V9.1: Protein-protein interaction networks, with increased coverage and integration. Nucleic Acids Res. 41, D808–D815. doi: 10.1093/nar/gks1094 23203871 PMC3531103

[B46] French E. Kim B. S. Iyer-Pascuzzi A. S. (2016). Mechanisms of quantitative disease resistance in plants. Semin. Cell Dev. Biol. 56, 201–208. doi: 10.1016/j.semcdb.2016.05.015 27212254

[B47] Gassmann W. Bhattacharjee S. (2012). Effector-triggered immunity signaling: from gene-for-gene pathways to protein-protein interaction networks. Mol. Plant Microbe Interact. 25, 862–868. doi: 10.1094/MPMI-01-12-0024-IA 22414439

[B48] Glazebrook J. (2005). Contrasting mechanisms of defense against biotrophic and necrotrophic pathogens. Annu. Rev. Phytopathol. 43, 205–227. doi: 10.1146/annurev.phyto.43.040204.135923 16078883

[B49] Gómez-Gómez L. Boller T. (2000). FLS2: An LRR receptor-like kinase involved in the perception of the bacterial elicitor flagellin in *Arabidopsis* . Mol. Cell 5, 1003–1011. doi: 10.1016/s1097-2765(00)80265-8 10911994

[B50] Graham T. L. Graham M. Y. Subramanian S. Yu O. (2007). RNAi silencing of genes for elicitation or biosynthesis of 5-deoxyisoflavonoids suppresses race-specific resistance and hypersensitive cell death in Phytophthora sojae infected tissues. Plant Physiol. 144, 728–740. doi: 10.1104/pp.107.097865 17416637 PMC1914209

[B51] Graham M. Y. Weidner J. Wheeler K. Pelow M. L. Graham T. L. (2003). Induced expression of pathogenesis-related protein genes in soybean by wounding and the *Phytophthora sojae* cell wall glucan elicitor. Mol. Plant Pathol. 63, 141–149. doi: 10.1016/j.pmpp.2003.11.002

[B52] Grau C. R. Dorrance A. E. Bond J. Russin J. S. (2004). “Fungal diseases,” in Soybeans: Improvement, Production, and Uses. Eds. Shibles R. M. Harper J. E. Wilson R. F. Shoemaker R. C. (Madison, WI: American Society of Agronomy, Inc), 679–763. doi: 10.2134/agronmonogr16.3ed.c14

[B53] Green B. F. Tukey J. W. (1960). Complex analyses of variance: general problems. Psychometrika 25, 127–152. doi: 10.1007/BF02288577

[B54] Guo H. Nolan T. M. Song G. Liu S. Xie Z. Chen J.. (2018). FERONIA receptor kinase contributes to plant immunity by suppressing jasmonic acid signaling in *Arabidopsis thaliana* . Curr. Biol. 28, 3316–3324. doi: 10.1016/j.cub.2018.07.078 30270181

[B55] Guo X. Wang D. Gordon S. G. Helliwell E. Smith T. Berry S. A.. (2008). Genetic mapping of QTLs underlying partial resistance to *Sclerotinia sclerotiorum* in soybean PI 391589A and PI 391589B. Crop Sci. 48, 1129–1139. doi: 10.2135/cropsci2007.04.0198

[B56] Hale B. Brown E. Wijeratne A. (2023a). An updated assessment of the soybean-*Phytophthora sojae* pathosystem. Plant Pathology. 72, 843–860. doi: 10.1111/ppa.13713

[B57] Hale B. Ratnayake S. Flory A. Wijeratne R. Schmidt C. Robertson A. E.. (2023b). Gene regulatory network inference in soybean upon infection by *Phytophthora sojae* . PLoS One 18, e0287590. doi: 10.1371/journal.pone.0287590 37418376 PMC10328377

[B58] Ham B. K. Li G. Kang B. H. Zeng F. Lucas W. J. (2012). Overexpression of *Arabidopsis* plasmodesmata germin-like proteins disrupts root growth and development. Plant Cell 24, 3630–3648. doi: 10.1105/tpc.112.101063 22960910 PMC3480292

[B59] Hammond J. P. Mayes S. Bowen H. C. Chram N. S. Hayden R. M. Love C. G.. (2011). Regulatory hotspots are associated with plant gene expression under varying soil phosphorus supply in *Brassica rapa* . Plant Physiol. 156, 1230–1241. doi: 10.1104/pp.111.175612 21527424 PMC3135916

[B60] Han Y. Teng W. Yu K. Poysa V. Anderson T. Qiu L.. (2008). Mapping QTL tolerance to Phytophthora root rot in soybean using microsatellite and RAPD/SCAR derived markers. Euphytica 162, 231–239. doi: 10.1007/s10681-007-9558-4

[B61] Hohmann U. Lau K. Hothorn M. (2017). The structural basis of ligand perception and signal activation by receptor kinases. Annu. Rev. Plant Biol. 68, 109–137. doi: 10.1146/annurev-arplant-042916-040957 28125280

[B62] Huang J. Guo N. Li Y. Sun J. Hu G. Zhang H.. (2016). Phenotypic evaluation and genetic dissection of resistance to *Phytophthora sojae* in the Chinese soybean mini core collection. BMC Genet. 17, 85. doi: 10.1186/s12863-016-0383-4 27316671 PMC4912746

[B63] Huber W. Carey V. J. Gentleman R. Anders S. Carlson M. Carvalho B. S.. (2015). Orchestrating high-throughput genomic analysis with Bioconductor. Nat. Methods 12, 115–121. doi: 10.1038/nmeth.3252 25633503 PMC4509590

[B64] Hubner N. Wallace C. A. Zimdahl H. Petretto E. Schulz H. Maciver F.. (2005). Integrated transcriptional profiling and linkage analysis for identification of genes underlying disease. Nat. Genet. 37, 243–253. doi: 10.1038/ng1522 15711544

[B65] Imholte G. C. Scott-Boyer M. P. Labbe A. Deschepper C. F. Gottardo R. (2013). iBMQ: R/Bioconductor package for integrated Bayesian modeling of eQTL data. Bioinformatics 29, 2797–2798. doi: 10.1093/bioinformatics/btt485 23958729 PMC3799478

[B66] Jahan M. A. Harris B. Lowery M. Infante A. M. Percifield R. J. Kovinich N. (2020). Glyceollin transcription factor GmMYB29A2 regulates soybean resistance to *Phytophthora sojae* . Plant Physiol. 183, 530–546. doi: 10.1104/pp.19.01293 32209590 PMC7271783

[B67] Jansen R. C. Nap J. P. (2001). Genetical genomics: The added value from segregation. Trends Genet. 17, 388–391. doi: 10.1016/S0168-9525(01)02310-1 11418218

[B68] Jansen R. C. Tesson B. M. Fu J. Yang Y. McIntyre L. M. (2009). Defining gene and QTL networks. Curr. Opin. Plant Biol. 12, 241–246. doi: 10.1016/j.pbi.2009.01.003 19196544

[B69] Jones J. D. Dangl J. L. (2006). The plant immune system. Nature 444, 323–329. doi: 10.1038/nature05286 17108957

[B70] Jun T. H. Mian M. A. R. Kang S. T. Michel A. P. (2012). Genetic mapping of the powdery mildew resistance gene in soybean PI 567301B. Theor. Appl. Genet. 125, 1159–1168. doi: 10.1007/s00122-012-1902-y 22692446

[B71] Kanehisa M. Goto S. (2000). KEGG: Kyoto encyclopedia of genes and genomes. Nucleic Acids Res. 28, 27–30. doi: 10.1093/nar/28.1.27 10592173 PMC102409

[B72] Karhoff S. Vargas-Garcia C. Lee S. Mian M. A. R. Graham M. A. Dorrance A. E.. (2022). Identification of candidate genes for a major quantitative disease resistance locus from soybean PI 427105B for resistance to *Phytophthora sojae* . Front. Plant Sci. 13. doi: 10.3389/fpls.2022.893652 PMC9237613 35774827

[B73] Kemmerling B. Halter T. Mazzotta S. Mosher S. Nürnberger T. (2011). A genome-wide survey for *Arabidopsis* leucine-rich repeat receptor kinases implicated in plant immunity. Front. Plant Sci. 2. doi: 10.3389/fpls.2011.00088 PMC3355784 22645555

[B74] Keurentjes J. J. B. Fu J. Terpstra I. R. Garcia J. M. van den Ackerveken G. Snoek L. B.. (2007). Regulatory network construction in *Arabidopsis* by using genome-wide gene expression quantitative trait loci. Proc. Natl. Acad. Sci. U.S.A. 104, 1708–1713. doi: 10.1073/pnas.0610429104 17237218 PMC1785256

[B75] Kidd B. N. Kadoo N. Y. Dombrecht B. Tekeoglu M. Gardiner D. M. Thatcher L. F.. (2011). Auxin signaling and transport promote susceptibility to the root-infecting fungal pathogen *Fusarium oxysporum* in *Arabidopsis* . Mol. Plant Microbe Interact. 24, 733–748. doi: 10.1094/MPMI-08-10-0194 21281113

[B76] Kim H. Diers B. (2000). Inheritance of partial resistance to Sclerotinia stem rot in soybean. Crop Sci. 40, 55–61. doi: 10.2135/cropsci2000.40155x

[B77] Kliebenstein D. (2009). Quantitative genomics: Analyzing intraspecific variation using global gene expression polymorphisms or eQTLs. Annu. Rev. Plant Biol. 60, 93–114. doi: 10.1146/annurev.arplant.043008.092114 19012536

[B78] Langfelder P. Horvath S. (2007). Eigengene networks for studying the relationships between co-expression modules. BMC Syst. Biol. 1, 1–17. doi: 10.1186/1752-0509-1-54 18031580 PMC2267703

[B79] Langfelder P. Horvath S. (2008). WGCNA: An R package for weighted correlation network analysis. BMC Bioinf. 9, 559. doi: 10.1186/1471-2105-9-559 PMC2631488 19114008

[B80] Lee H. Y. Bowen C. H. Popescu G. V. Kang H.-G. Kato N. Ma S.. (2011). *Arabidopsis* RTNLB1 and RTNLB2 reticulon-like proteins regulate intracellular trafficking and activity of the FLS2 immune receptor. Plant Cell. 9, 3374–3391. doi: 10.1105/tpc.111.089656 PMC3203430 21949153

[B81] Lee S. Mian M. A. R. McHale L. K. Sneller C. H. Dorrance A. E. (2013a). Identification of quantitative trait loci conditioning partial resistance to Phytophthora sojae in soybean PI 407861A. Crop Sci. 53, 1022–1031. doi: 10.2135/cropsci2012.10.0578 PMC3607739 23354974

[B82] Lee S. Mian M. A. R. McHale L. K. Wang H. Wijeratne A. J. Sneller. (2013b). Novel quantitative trait loci for partial resistance to *Phytophthora sojae* in soybean PI 398841. Theor. Appl. Genet. 126, 1121–1132. doi: 10.1007/s00122-013-2040-x 23354974 PMC3607739

[B83] Lee S. Mian M. A. R. Sneller C. H. Wang H. Dorrance A. E. McHale L. K. (2014). Joint linkage QTL analyses for partial resistance to *Phytophthora sojae* in soybean using six nested inbred populations with heterogeneous conditions. Theor. Appl. Genet. 127, 429–444. doi: 10.1007/s00122-013-2229-z 24247235

[B84] Li Z. (2019). “Molecular analysis of epistasis affecting complex traits,” in Molecular Dissection of Complex Traits. Ed. Patterson A. H. (Boca Raton, FL: CRC Press), 119–130. doi: 10.1201/9780429117770-8

[B85] Li X. Han Y. Teng W. Zhang A. Yu K. Poysa V.. (2010). Pyramided QTL underlying tolerance to Phytophthora root rot in mega-environments from soybean cultivars “Conrad” and “Hefeng 25”. Theor. Appl. Genet. 121, 651–658. doi: 10.1007/s00122-010-1337-2 20390244

[B86] Li R. Jeong K. Davis J. T. Kim S. Lee S. Michelmore R. W.. (2018). Integrated QTL and eQTL mapping provides insights and candidate genes for fatty acid composition, flowering time, and growth traits in a F2 population of a novel synthetic allopolyploid *Brassica napus* . Front. Plant Sci. 9. doi: 10.3389/fpls.2018.01632 PMC6243938 30483289

[B87] Liao Y. Smyth G. K. Shi W. (2014). featureCounts: An efficient general purpose program for assigning sequence reads to genomic features. Bioinformatics 30, 923–930. doi: 10.1093/bioinformatics/btt656 24227677

[B88] Lin F. Chhapekar S. S. Vieira C. C. Da Silva M. P. Rojas A. Lee D.. (2022). Breeding for disease resistance in soybean: a global perspective. Theor. Appl. Gen. 135 (11), 3773–3872. doi: 10.1007/s00122-022-04101-3 PMC9729162 35790543

[B89] Lin F. Zhao M. Baumann D. D. Ping J. Sun L. Liu Y.. (2014). Molecular response to the pathogen *Phytophthora sojae* among ten soybean near isogenic lines revealed by comparative transcriptomics. BMC Genomics 15, 18. doi: 10.1186/1471-2164-15-18 24410936 PMC3893405

[B90] Liu Q. Liu H. Gong Y. Tao Y. Jiang L. Zuo W.. (2017). An atypical thioredoxin imparts early resistance to *Sugarcane mosaic virus* in maize. Mol. Plant 10, 483–497. doi: 10.1016/j.molp.2017.02.002 28216424

[B91] Liu J. Osbourn A. Ma P. (2015). MYB transcription factors regulators of phenylpropanoid metabolism in plants. Mol. Plant 8, 689–708. doi: 10.1016/j.molp.2015.03.012 25840349

[B92] Lorenzo O. Piqueras R. Sanchez-Serrano J. J. Solano R. (2003). Ethylene response factor 1 integrates signals from ethylene and jasmonate pathways in plant defense. Plant Cell 15, 165–178. doi: 10.1105/tpc.007468 12509529 PMC143489

[B93] Lygin A. V. Zernova O. V. Hill C. B. Kholina N. A. Widholm J. M. Hartman G. L.. (2013). Glyceollin is an important component of soybean plant defense against *Phytophthora sojae* and *Macrophomina phaseolina* . Phytopathology 103, 984–994. doi: 10.1094/PHYTO-12-12-0328-R 23617338

[B94] MacDonald K. L. Cahill D. M. (1999). Influence of abscisic acid and the abscisic acid biosynthesis inhibitor, norflurazon, on interactions between *Phytophthora sojae* and soybean (*Glycine max*). Eur. J. Plant Pathol. 60, 185–195. doi: 10.1023/A:1008705321113

[B95] Mideros S. Nita M. Dorrance A. E. (2007). Characterization of components of partial resistance, *Rps2*, and root resistance to *Phytophthora sojae* in soybean. Phytopathology 97, 655–662. doi: 10.1094/PHYTO-97-5-0655 18943586

[B96] Minic Z. (2008). Physiological roles of plant glycoside hydrolases. Planta 227, 723–740. doi: 10.1007/s00425-007-0668-y 18046575

[B97] Mohr P. G. Cahill D. M. (2001). Relative roles of glyceollin, lignin and the hypersensitive response and the influence of ABA incompatible and incompatible interactions of soybeans with *Phytophthora sojae* . Physiol. Mol. Plant Pathol. 58, 31–41. doi: 10.1006/pmpp.2000.0306

[B98] Moscou M. J. Lauter N. Steffenson B. Wise R. P. (2011). Quantitative and qualitative stem rust resistance factors in barley are associated with transcriptional suppression of defense regulons. PLoS Genet. 7, e1002208. doi: 10.1371/journal.pgen.1002208 21829384 PMC3145622

[B99] Moy P. Qutob D. Chapman B. P. Atkinson I. Gijzen M. (2004). Patterns of gene expression upon infection of soybean plants by *Phytophthora sojae* . Mol. Plant Microbe Interact. 17, 1051–1062. doi: 10.1094/MPMI.2004.17.10.1051 15497398

[B100] Naveed Z. A. Wei X. Chen J. Mubeen H. Ali G. S. (2020). The PTI to ETI continuum in *Phytophthora*-plant interactions. Front. Plant Sci. 11. doi: 10.3389/fpls.2020.593905 PMC7773600 33391306

[B101] Nelson R. Wiesner-Hanks T. Wisser R. Balint-Kurti P. (2018). Navigating complexity to breed disease-resistant crops. Nat. Rev. Genet. 19, 21–33. doi: 10.1038/nrg.2017.82 29109524

[B102] Neto E. C. Keller M. P. Broman A. F. Attie A. D. Jansen R. C. Broman K. W.. (2012). Quantile-based permutation thresholds for quantitative trait loci hotspots. Genetics 191, 1355–1365. doi: 10.1534/genetics.112.139451 22661325 PMC3416013

[B103] Ng D. K. Abeysinghe J. K. Kamali M. (2018). Regulating the regulators: The control of transcription factors in plant defense signaling. Int. J. Mol. Sci. 19, 3737. doi: 10.3390/ijms19123737 30477211 PMC6321093

[B104] Nguyen V. T. Vuong T. D. VanToai T. Lee J. D. Wu X. Rouf Mian M. A.. (2012). Mapping of quantitative trait loci associated with resistance to *Phytophthora sojae* and flooding tolerance in soybean. Crop Sci. 52, 2481–2493. doi: 10.2135/cropsci2011.09.0466

[B105] Niks R. E. Qi X. Q. Marcel T. C. (2015). Quantitative resistance to biotrophic filamentous plant pathogens: concepts, misconceptions, and mechanisms. Annu. Rev. Phytopathol. 53, 445–470. doi: 10.1146/annurev-phyto-080614-115928 26047563

[B106] Pieterse C. Leon-Reyes A. van der Ent S. Van Wees S. (2009). Networking by small-molecule hormones in plant immunity. Nat. Chem. Biol. 5, 308–316. doi: 10.1038/nchembio.164 19377457

[B107] Pieterse C. M. van der Does D. Zamioudis C. Leon-Reyes A. Van Wees S. C. (2012). Hormonal modulation of plant immunity. Ann. Rev. Cell Dev. Biol. 28, 489–521. doi: 10.1146/annurev-cellbio-092910-154055 22559264

[B108] Pilet-Nayel M. L. Moury B. Caffier V. Montarry J. Kerlan M. C. Fournet S.. (2017). Quantitative resistance to plant pathogens in pyramiding strategies for durable crop protection. Front. Plant Sci. 8. doi: 10.3389/fpls.2017.01838 PMC5664368 29163575

[B109] Poland J. A. Balint-Kurti P. J. Wisser R. J. Pratt R. C. Nelson R. J. (2009). Shades of gray: The world of quantitative disease resistance. Trends Plant Sci. 14, 21–29. doi: 10.1016/j.tplants.2008.10.006 19062327

[B110] Poovaiah B. W. Reddy A. S. (1993). Calcium and signal transduction in plants. CRC Crit. Rev. Plant Sci. 12, 185–211. doi: 10.1080/07352689309701901 11540065

[B111] Potokina E. Druka A. Luo Z. Wise R. Waugh R. Kearsey M. (2008). Gene expression quantitative trait locus analysis of 16,000 barley genes reveals a complex pattern of genome-wide transcriptional regulation. Plant J. 53, 90–101. doi: 10.1111/j.1365-313X.2007.03315.x 17944808

[B112] Qu W. Gurdziel K. Pique-Regi R. Ruden D. M. (2018). Lead modulated trans- and cis- expression quantitative trait loci (eQTLs) in *Drosophila melanogaster* heads. Front. Genet. 9. doi: 10.3389/fgene.2018.00395 PMC6158337 30294342

[B113] Ranathunge K. Thomas R. H. Fang X. Peterson C. A. Gijzen M. Bernards M. A. (2008). Soybean root suberin and partial resistance to root rot caused by *Phytophthora sojae* . Phytopathology 98, 1179–1189. doi: 10.1094/PHYTO-98-11-1179 18943406

[B114] R Core Team. (2018). R: A language and environment for statistical computing (Vienna, Austria: R Foundation for Statistical Computing). Available at: https://www.R-project.org/.

[B115] Reddy A. S. N. Reddy V. (2001). “Calcium as a messenger in stress signal transduction,” in Handbook of Plant and Crop Physiology. Ed. Pessarakali M. (Boca Raton, FL: CRC Press), 697–732. doi: 10.1201/9780203908426.ch35

[B116] Rehman S. Mahmood T. (2015). Functional role of DREB and ERF transcription factors: regulating stress-responsive network in plants. Acta Physiol. Plant 37, 1–14. doi: 10.1007/s11738-015-1929-1

[B117] Rincker K. Hartman G. L. Diers B. W. (2016). Fine mapping of resistance genes from five brown stem rot resistance sources in soybean. Plant Genome 9. doi: 10.3835/plantgenome2015.08.0063 27898763

[B118] Robinson M. D. McCarthy D. J. Smyth G. K. (2010). edgeR: A Bioconductor package for differential expression analysis of digital gene expression data. Bioinformatics 26, 139–140. doi: 10.1093/bioinformatics/btp616 19910308 PMC2796818

[B119] Rolling W. Lake R. Dorrance A. E. McHale L. K. (2020). Genome-wide association analyses of quantitative disease resistance in diverse sets of soybean [*Glycine max* (L.) Merr.] plant introductions. PLoS One 15, e0227710. doi: 10.1371/journal.pone.0227710 32196522 PMC7083333

[B120] Roux F. Voisin D. Badet T. Balagué C. Barlet X. Huard-Chauveau C.. (2014). Resistance to phytopathogens *e tutti quanti*: placing plant quantitative disease resistance on the map. Mol. Plant Pathol. 15, 427–432. doi: 10.1111/mpp.12138 24796392 PMC6638617

[B121] Saha A. Battle A. (2018). False positives in trans-eQTL and co-expression analyses arising from RNA-sequencing alignment errors. F1000Research 7, 1860. doi: 10.12688/f1000research.17145.2 30613398 PMC6305209

[B122] Salvi S. Tuberosa R. (2005). To clone or not to clone plant QTLs: Present and future challenges. Trends Plant Sci. 10, 297–304. doi: 10.1016/j.tplants.2005.04.008 15949764

[B123] Samad-Zamini M. Schweiger W. Nussbaumer T. Mayer K. F. X. Buerstmayr H. (2017). Time-course expression QTL atlas of the global transcriptional response of wheat to *Fusarium graminearum* . Plant Biotechnol. J. 15, 1453–1464. doi: 10.1111/pbi.12729 28332274 PMC5633761

[B124] Schadt E. E. Monks S. A. Drake T. A. Lusis A. J. Che N. Colinayo V.. (2003). Genetics of gene expression surveyed in maize, mouse and man. Nature 422, 297–302. doi: 10.1038/nature01434 12646919

[B125] Schmitthenner A. F. (1985). Problems and progress in control of Phytophthora root rot of soybean. Plant Dis. 69, 362–368. doi: 10.1094/PD-69-362

[B126] Schmutz J. Cannon S. B. Schlueter J. Ma J. Mitros T. Nelson W.. (2010). Genome sequence of the paleopolyploid soybean. Nature 463, 178–183. doi: 10.1038/nature08670 20075913

[B127] Schneider R. Rolling W. Song Q. Cregan P. Dorrance A. E. McHale L. K. (2016). Genome-wide association mapping of partial resistance to *Phytophthora sojae* in soybean plant introductions from the Republic of Korea. BMC Genomics 17, 607. doi: 10.1186/s12864-016-2918-5 27515508 PMC4982113

[B128] Scott K. Balk C. Veney D. McHale L. K. Dorrance A. E. (2019). Quantitative disease resistance loci towards *Phytophthora sojae* and three species of *Pythium* in six soybean nested association mapping populations. Crop Sci. 59, 605–623. doi: 10.2135/cropsci2018.09.0573

[B129] Shiu S. H. Karlowski W. M. Pan R. Tzeng Y. H. Mayer K. F. Li W. H. (2004). Comparative analysis of the receptor-like kinase family in *Arabidopsis* and rice. Plant Cell 16, 1220–1234. doi: 10.1105/tpc.020834 15105442 PMC423211

[B130] Smith C. J. Watson C. F. Morris P. C. Bird C. R. Seymour G. B. Gray J. E.. (1990). Inheritance and effect on ripening of antisense polygalacturonase genes in transgenic tomatoes. Plant Molec. Biol. 14 (3), 369–379. doi: 10.1007/BF00028773 2102820

[B131] Smith C. J. S. Watson C. F. Ray J. Bird C. R. Morris P. C. Schuch W.. (1988). Antisense RNA inhibition of polygalacturonase gene expression in transgenic tomatoes. Nature 334, 724–726. doi: 10.1038/334724a0

[B132] Soltis N. E. Caseys C. Zhang W. Corwin J. A. Atwell S. Kliebenstein D. J. (2020). Pathogen genetic control of transcriptome variation in the *Arabidopsis thaliana* – *Bortrytis cinerea* pathosystem. Genetics 215, 253–266. doi: 10.1534/genetics.120.303070 32165442 PMC7198280

[B133] Soyars C. L. James S. R. Nimchuk Z. L. (2016). Ready, aim, shoot: Stem cell regulation of the shoot apical meristem. Curr. Opin. Plant Biol. 29, 163–168. doi: 10.1016/j.pbi.2015.12.002 26803586

[B134] Spoel S. H. Dong X. (2012). How do plants achieve immunity? Defence without specialized immune cells. Nat. Rev. Immunol. 12, 89–100. doi: 10.1038/nri3141 22273771

[B135] Stasko A. K. Batnini A. Bolanos-Carriel C. Lin J. E. Lin Y. Blakeslee J.. (2020). Auxin profiling and *GmPIN* expression in *Phytophthora sojae*-soybean root interactions. Phytopathology 110, 1988–2002. doi: 10.1094/PHYTO-02-20-0046-R 32602813

[B136] Stasko A. K. Wickramasinghe D. Nauth B. J. Acharya B. Ellis M. L. Taylor C. G.. (2016). High-density mapping of resistance QTL toward *Phytophthora sojae*, *Pythium irregulare*, and *Fusarium graminearum* in the same soybean population. Crop Sci. 56, 2476–2492. doi: 10.2135/cropsci2015.12.0749

[B137] St. Clair D. A. (2010). Quantitative disease resistance and quantitative resistance loci in breeding. Annu. Rev. Phytopathol. 48, 247–268. doi: 10.1146/annurev-phyto-080508-081904 19400646

[B138] Stracke R. (2001). The R2R3-MYB gene family in *Arabidopsis thaliana.* Curr. Opin. Plant Biol. 4, 447–456. doi: 10.1016/S1369-5266(00)00199-0 11597504

[B139] Sugano S. Sugimoto T. Takatsuji H. Jiang C. J. (2013). Induction of resistance to *Phytophthora sojae* in soyabean (*Glycine max*) by salicylic acid and ethylene. Plant Pathol. 62, 1048–1056. doi: 10.1111/ppa.12011

[B140] Sun Y. Wu Y. Yang C. Sun S. Lin X. Liu L.. (2017). Segmental allotetraploidy generates extensive homoeologous expression rewiring and phenotypic diversity at the population level in rice. Mol. Ecol. 26, 5451–5466. doi: 10.1111/mec.14297 28802080

[B141] Swanson S. Gilroy S. (2010). ROS in plant development. Physiol. Plant 138 (4), 384–392. doi: 10.1111/j.1399-3054.2009.01313.x 19947976

[B142] Swanson-Wagner R. A. DeCook R. Jia Y. Bancroft T. Ji T. Zhao X.. (2009). Paternal dominance of trans-eQTL influences gene expression patterns in maize hybrids. Science 326, 1118–1120. doi: 10.1126/science.1178294 19965432

[B143] Thomas R. Fang X. Ranathunge K. Anderson T. R. Peterson C. A. Bernards M. A. (2007). Soybean root suberin: Anatomical distribution, chemical composition, and relationship to partial resistance to *Phytophthora sojae* . Plant Physiol. 144, 299–311. doi: 10.1104/pp.106.091090 17494920 PMC1913776

[B144] Tian J. Keller M. P. Broman A. T. Kendziorski C. Yandell B. S. Attie A. D.. (2016). The dissection of expression quantitative trait locus hotspots. Genetics 202, 1563–1574. doi: 10.1534/genetics.115.183624 26837753 PMC4905536

[B145] Tian T. Liu Y. Yan H. You Q. Yi X. Du Z.. (2017). agriGO v2.0: A GO analysis toolkit for the agricultural community 2017 update. Nucleic Acids Res. 45, W122–W129. doi: 10.1093/nar/gkx382 28472432 PMC5793732

[B146] Tooley P. W. Grau C. (1982). Identification and quantitative characterization of rate-reducing resistance to *Phytophthora megasperma* f.sp. *glycinea* in soybean seedlings. Phytopathology 72, 727–733. doi: 10.1094/Phyto-72-727

[B147] Torres M. A. (2010). ROS in biotic interactions. Physiol. Plant 138 (4), 414–429. doi: 10.1111/j.1399-3054.2009.01326.x 20002601

[B148] Trewavas A. J. Mahlo R. (1998). Ca^2+^ signaling in plant cells: The big network! Curr. Opin. Plant Biol. 1, 428–433. doi: 10.1016/S1369-5266(98)80268-9 10066614

[B149] Tucker D. M. Saghai Maroof M. A. Mideros S. Skoneczka J. A. Nabati D. A. Buss G. R.. (2010). Mapping quantitative trait loci for partial resistance to *Phytophthora sojae* in a soybean interspecific cross. Crop Sci. 50, 628–635. doi: 10.2135/cropsci2009.03.0161

[B150] Vailleau F. Daniel X. Tronchet M. Montillet J. L. Triantaphylides C. Roby D. (2002). A R2R3-MYB gene, AtMYB30, acts as a positive regulator of the hypersensitive cell death program in plants in response to pathogen attack. Proc. Natl. Acad. Sci. U.S.A. 99, 10179–10184. doi: 10.1073/pnas.152047199 12119395 PMC126644

[B151] Vega-Sánchez M. Redinbaugh M. Costanzo S. Dorrance A. E. (2005). Spatial and temporal expression analysis of defense-related genes in soybean cultivars with different levels of partial resistance to *Phytophthora sojae* . Physio. Mol. Plant Pathol. 66, 175–182. doi: 10.1016/j.pmpp.2005.07.001

[B152] Vuong T. D. Diers B. W. Hartman G. L. (2008). Identification of QTL for resistance to Sclerotinia stem rot in soybean plant introduction 194639. Crop Sci. 48, 2209–2214. doi: 10.2135/cropsci2008.01.0019

[B153] Walton J. D. (1994). Deconstructing the cell wall. Plant Physiol. 104, 1113–1118. doi: 10.1104/pp.104.4.1113 12232152 PMC159271

[B154] Wang X. Chen Q. Wu Y. Lemmon Z. H. Xu G. Huang C.. (2017). Genome-wide analysis of transcriptional variability in large maize-teosinte population. Mol. Plant 11, 443–459. doi: 10.1016/j.molp.2017.12.011 29275164

[B155] Wang Y. Han Y. Teng W. Zhao X. Li Y. Wu L.. (2014). Expression quantitative trait loci infer the regulation of isoflavone accumulation in soybean (*Glycine max* L. Merr.) seed. BMC Genomics 15, 1–11. doi: 10.1186/1471-2164-15-680 25124843 PMC4138391

[B156] Wang H. St. Martin S. K. Dorrance A. E. (2012a). Comparison of phenotypic methods and yield contributions of quantitative trait loci for partial resistance to Phytophthora sojae in soybean. Crop Sci. 52, 1–14. doi: 10.2135/cropsci2011.06.0336

[B157] Wang Y. Tyler B. M. Wang Y. (2019). Defense and counter defense during plant-pathogenic oomycete infection. Annu. Rev. Microbiol. 73, 667–696. doi: 10.1146/annurev-micro-020518-120022 31226025

[B158] Wang H. Waller L. Tripathy S. St. Martin S. K. Zhou L. Krampis K.. (2010). Analysis of genes underlying soybean quantitative trait loci conferring partial resistance to *Phytophthora sojae* . Plant Genome J. 3, 23–40. doi: 10.3835/plantgenome2009.12.0029

[B159] Wang H. Wijeratne A. Wijeratne S. Lee S. Taylor C. G. St. Martin S. K.. (2012b). Dissection of two soybean QTL conferring partial resistance to *Phytophthora sojae* through sequence and gene expression analysis. BMC Genomics 13, 428. doi: 10.1186/1471-2164-13-428 22925529 PMC3443417

[B160] Wang S. Zheng T. Wang Y. (2007). Transcription activity hot spot, is it real or an artifact? BMC Proc. 1, S94. doi: 10.1186/1753-6561-1-S1-S94 18466598 PMC2367508

[B161] Ward E. W. B. Cahill D. N. Bhattacharyya M. (1989). Abscisic acid suppression of phenylalanine ammonia lyase activity and mRNA, and resistance of soybeans to *Phytophthora megasperma* f. sp. *glycinea* . Plant Physiol. 91, 23–27. doi: 10.1104/pp.91.1.23 16667002 PMC1061945

[B162] Weng C. Yu K. Anderson T. R. Poysa V. (2007). A quantitative trait locus influencing tolerance to Phytophthora root rot in the soybean cultivar “Conrad”. Euphytica 158, 81–86. doi: 10.1007/s10681-007-9428-0

[B163] Werner E. (2004). GTPases and reactive oxygen species: switches for killing and signaling. J. Cell Sci. 117 (2), 143–153. doi: 10.1242/jcs.00937 14676270

[B164] West M. A. L. Kim K. Kliebenstein D. J. van Leeuwnen H. Michelmore R. W. Doerge R. W.. (2007). Global eQTL mapping reveals the complex genetic architecture of transcript-level variation in *Arabidopsis* . Genetics 175, 1441–1450. doi: 10.1534/genetics.106.064972 17179097 PMC1840073

[B165] Wong J. Gao L. Yang Y. Zhai J. Arikit S. Yu Y.. (2014). Roles of small RNAs in soybean defense against *Phytophthora sojae* infection. Plant J. 79, 928–940. doi: 10.1111/tpj.12590 24944042 PMC5137376

[B166] Wu X. Blake S. Sleper D. A. Shannon J. G. Cregan P. Nguyen H. T. (2009). QTL, additive and epistatic effects for SCN resistance in PI 437654. Theor. Appl. Genet. 118, 1093–1105. doi: 10.1007/s00122-009-0965-x 19184662

[B167] Wu X. Zhou B. Zhao J. Guo N. Zhang B. Yang F.. (2011). Identification of quantitative trait loci for partial resistance to *Phytophthora sojae* in soybean. Plant Breed 130, 144–149. doi: 10.1111/j.1439-0523.2010.01799.x

[B168] Xu P. Wu J. Xue A. Li W. B. Chen W. Y. Wei L.. (2012). Differentially expressed genes of soybean during infection by *Phytophthora sojae.* J. Integr. Agric. 11, 368–377. doi: 10.1016/S2095-3119(12)60021-5

[B169] Yamaguchi Y. Huffaker A. Bryan A. C. Tax F. E. Ryan C. A. (2010). PEPR2 is a second receptor for the Pep1 and Pep2 peptides and contributes to defense responses in *Arabidopsis* . Plant Cell 22, 508–522. doi: 10.1105/tpc.109.068874 20179141 PMC2845411

[B170] Yan Q. Si J. Cui X. Peng H. Chen X. Xing H.. (2019). The soybean cinnamate 4-hydroxylase gene *GmC4H1* contributed positively to plant defense *via* increasing lignin content. Plant Growth Regul. 88, 139–149. doi: 10.1007/s10725-019-00494-2

[B171] Yao L. Berman B. P. Farnham P. J. (2015). Demystifying the secret mission of enhancers: Linking distal regulatory elements to target genes. Crit. Rev. Biochem. Mol. Biol. 50, 550–573. doi: 10.3109/10409238.2015.1087961 26446758 PMC4666684

[B172] Young N. D. (1996). QTL mapping and quantitative disease resistance in plants. Annu. Rev. Phytopathol. 34, 479–501. doi: 10.1146/annurev.phyto.34.1.479 15012553

[B173] Yuan N. Yuan S. Li Z. Zhou M. Wu P. Hu Q.. (2018). STRESS INDUCED FACTOR 2, a leucine-rich repeat kinase regulates basal plant pathogen defense. Plant Physiol. 176, 3062–3080. doi: 10.1104/pp.17.01266 29463771 PMC5884590

[B174] Zeng H. Zhang Y. Zhang X. Pi E. Zhu Y. (2017). Analysis of EF-hand proteins in soybean genome suggests their potential roles in environmental and nutritional stress signaling. Front. Plant Sci. 8. doi: 10.3389/fpls.2017.00877 PMC5443154 28596783

[B175] Zhang B. Horvath S. (2005). A general framework for weighted gene co-expression network analysis. Stat. Appl. Genet. Mol. Biol. 4. doi: 10.2202/1544-6115.1128 16646834

[B176] Zhang C. Wang X. Zhang F. Dong L. Wu J. Cheng Q.. (2017). Phenylalanine ammonia-lyase2.1 contributes to the soybean response towards *Phytophthora sojae* infection. Sci. Rep. 7, 7242. doi: 10.1038/s41598-017-07832-2 28775360 PMC5543151

[B177] Zhao Y. Chang X. Qi D. Dong L. Wang G. Fan S.. (2017). A novel soybean ERF transcription factor, GmERF113, increases resistance to *Phytophthora sojae* infection in soybean. Front. Plant Sci. 8. doi: 10.3389/fpls.2017.00299 PMC5339286 28326092

[B178] Zhao X. Han Y. Li Y. Lui D. Sun M. Zhao Y.. (2015). Loci and candidate gene identification to *Sclerotinia sclerotiorum* in soybean (*Glycine max* L. Merr.) *via* association and linkage maps. Plant J. 82, 245–255. doi: 10.1111/tpj.12810 25736370

[B179] Zhou L. Mideros S. X. Bao L. Hanlon R. Arredondo F. D. Tripathy S.. (2009). Infection and genotype remodel the entire soybean transcriptome. BMC Genomics 10, 49. doi: 10.1186/1471-2164-10-49 19171053 PMC2662884

[B180] Zielinski R. E. (1998). Calmodulin and calmodulin-binding proteins in plants. Ann. Rev. Physiol. Mol. Biol. 49, 697–725. doi: 10.1146/annurev.arplant.49.1.697 15012251

[B181] Zipfel C. (2014). Plant pattern-recognition receptors. Trends Immunol. 35, 345–251. doi: 10.1016/j.it.2014.05.004 24946686

[B182] Zipfel C. Kunze G. Chinchilla D. Caniard A. Jones J. D. Boller T.. (2006). Perception of the bacterial PAMP EF-Tu by the receptor EFR restricts Agrobacterium-mediated transformation. Cell 125, 749–760. doi: 10.1016/j.cell.2006.03.037 16713565

[B183] Zipfel C. Oldroyd G. E. D. (2017). Plant signaling in symbiosis and immunity. Nature 543, 328–336. doi: 10.1038/nature22009 28300100

