# Supplementary material for: Molecular mechanisms underpinning quantitative resistance to Phytophthora sojae in Glycine max using a systems genomics approach
Source: Front Plant Sci. 2023 Nov 7;14:1277585. doi: 10.3389/fpls.2023.1277585 (PMC10662313; doi:10.3389/fpls.2023.1277585)
Supplement: Supplementary file 2 [file DataSheet_2.pdf]

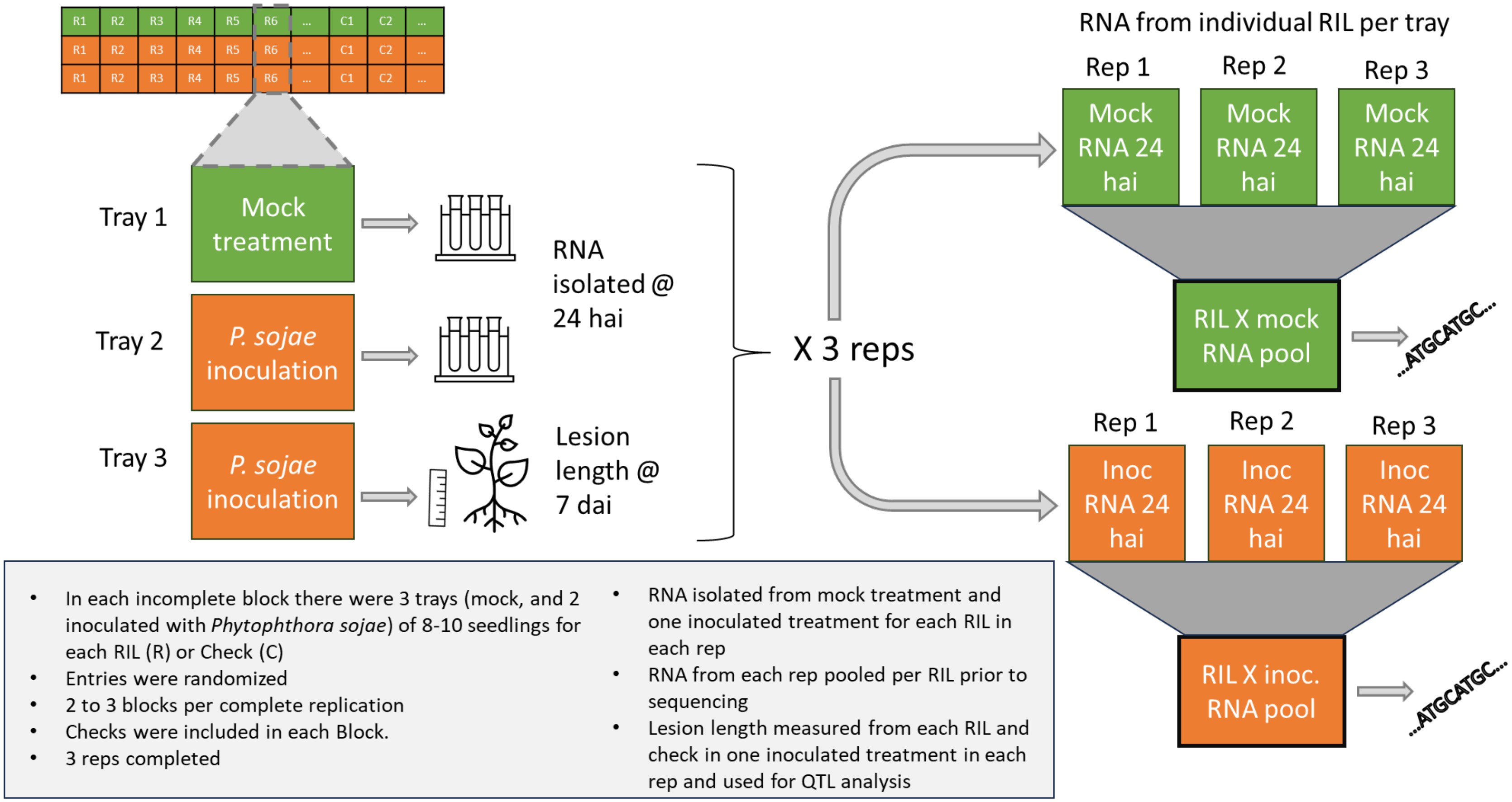

- In each incomplete block there were 3 trays (mock, and 2 inoculated with *Phytophthora sojae*) of 8-10 seedlings for each RIL (R) or Check (C)
- Entries were randomized
- 2 to 3 blocks per complete replication
- Checks were included in each Block.
- 3 reps completed
- RNA isolated from mock treatment and one inoculated treatment for each RIL in each rep
- RNA from each rep pooled per RIL prior to sequencing
- Lesion length measured from each RIL and check in one inoculated treatment in each rep and used for QTL analysis

Figure S1. Schematic for experimental design for incomplete blocks & RNA samples

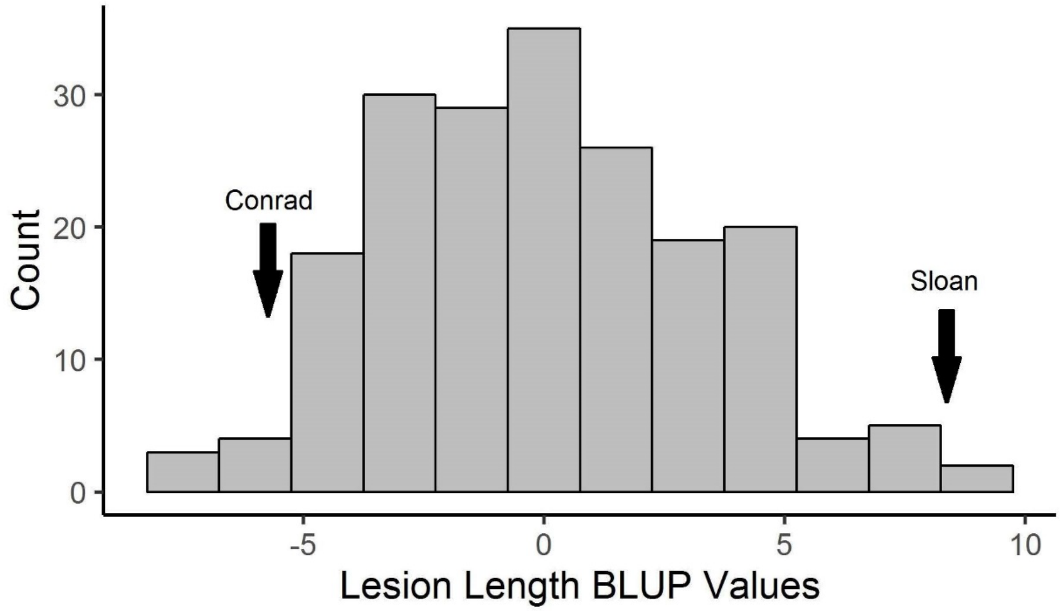

Figure S2. BLUP histogram for the Conrad x Sloan recombinant inbred line (RIL) subpopulation

Frequency distribution of best linear unbiased predictor (BLUP) values for mean lesion lengths of 93 RILs randomly selected from Conrad x Sloan F9:11 mapping population. A smaller BLUP value indicates a higher level of resistance to *Phytophthora sojae*. Arrows indicate best linear unbiased estimator (BLUE) values for parental lines Conrad (-6.70) and Sloan (8.20).

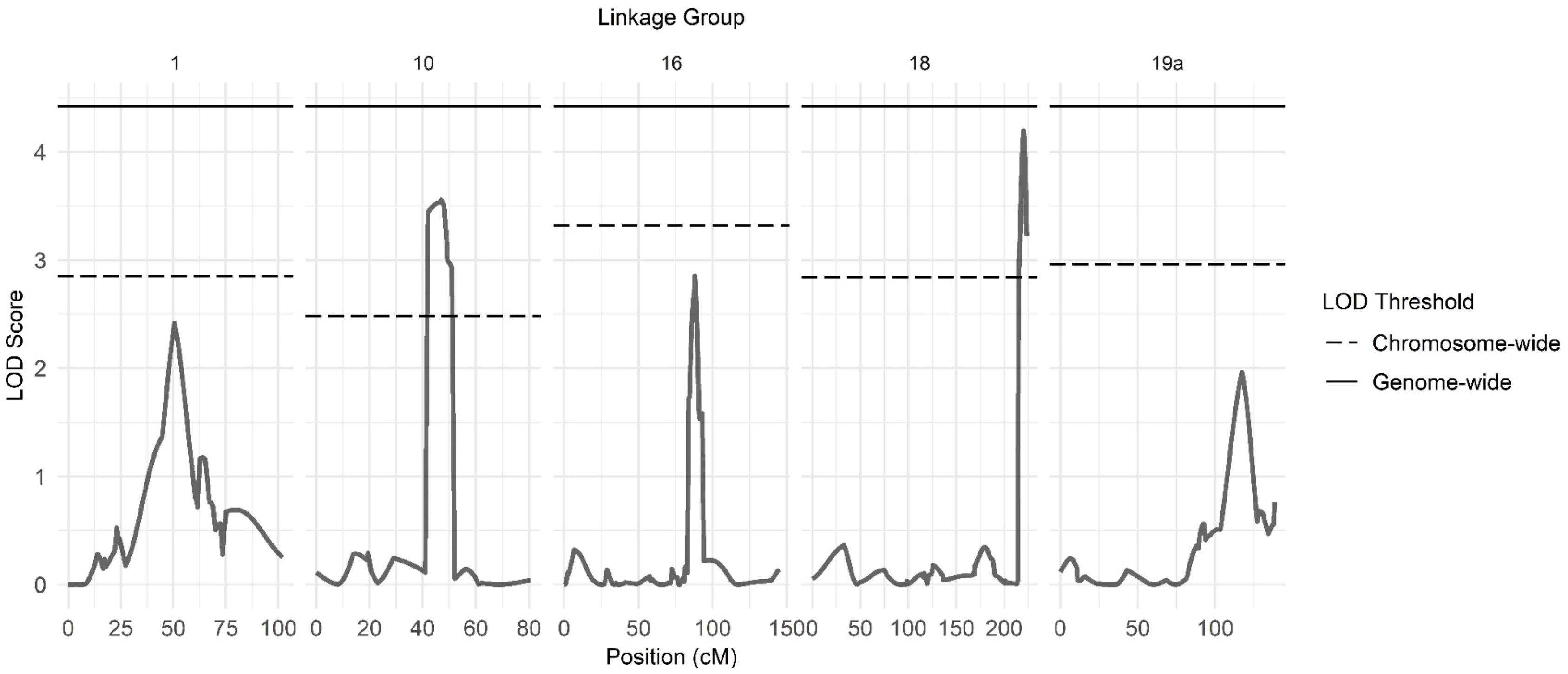

Figure S3. CIM mapping representing QTL peaks for subpopulation

Quantitative trait loci conferring resistance to *Phytophthora sojae* isolate 1.S.1.1 mapped in subset of Conrad x Sloan F9:11 recombinant inbred population with a genome-wide logarithm of odds (LOD) threshold of 4.42 for BLUP values obtained in present. Dashed lines indicate chromosome-wide LOD threshold, and solid line indicates genome-wide LOD threshold.

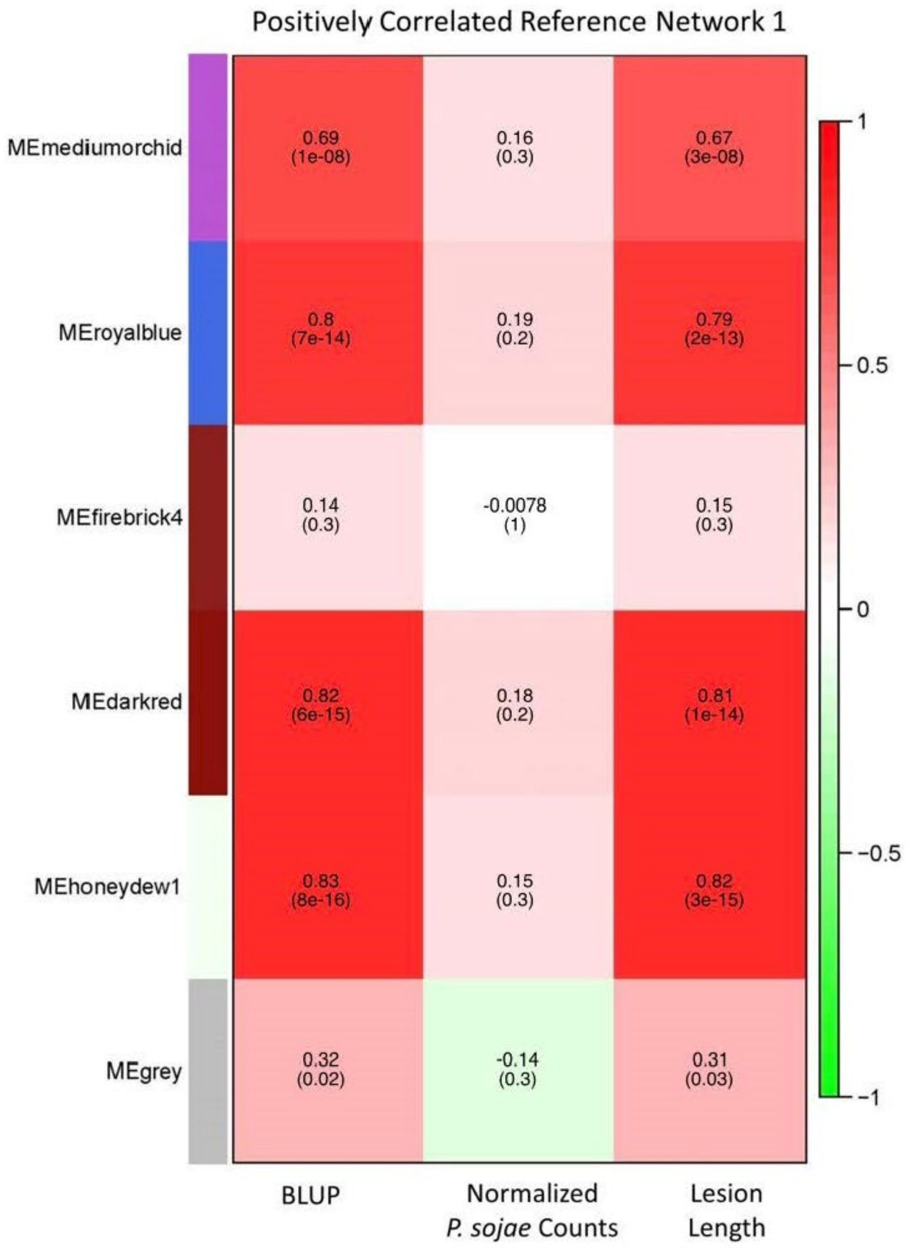

Figure S4. WGCNA representing reference network 1

Weighted gene co-expression analysis (WGCNA) reference network analysis 1 of modules that are positively correlated to BLUPs, lesion length values and normalized total *P. sojae* counts. Scale indicates correlation coefficient positive 1 (red) to negative 1 (green). Number in parenthesis is adjusted P-value, α=0.05.

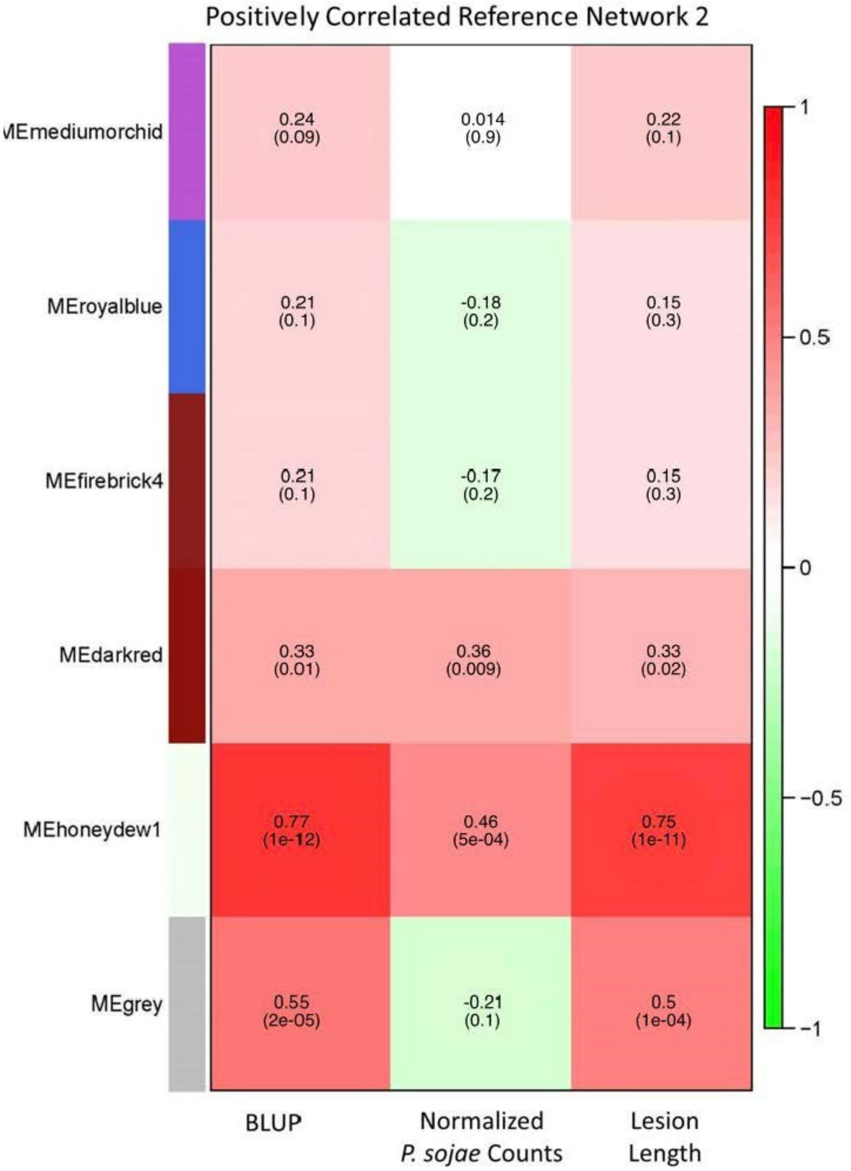

Figure S5. WGCNA representing reference network 2

Weighted gene co-expression analysis (WGCNA) reference network analysis 2 of modules that are positively correlated to BLUPs, lesion length values and normalized total *P. sojae* counts. Scale indicates correlation coefficient positive 1 (red) to negative 1 (green). Number in parenthesis is adjusted P-value, α=0.05.

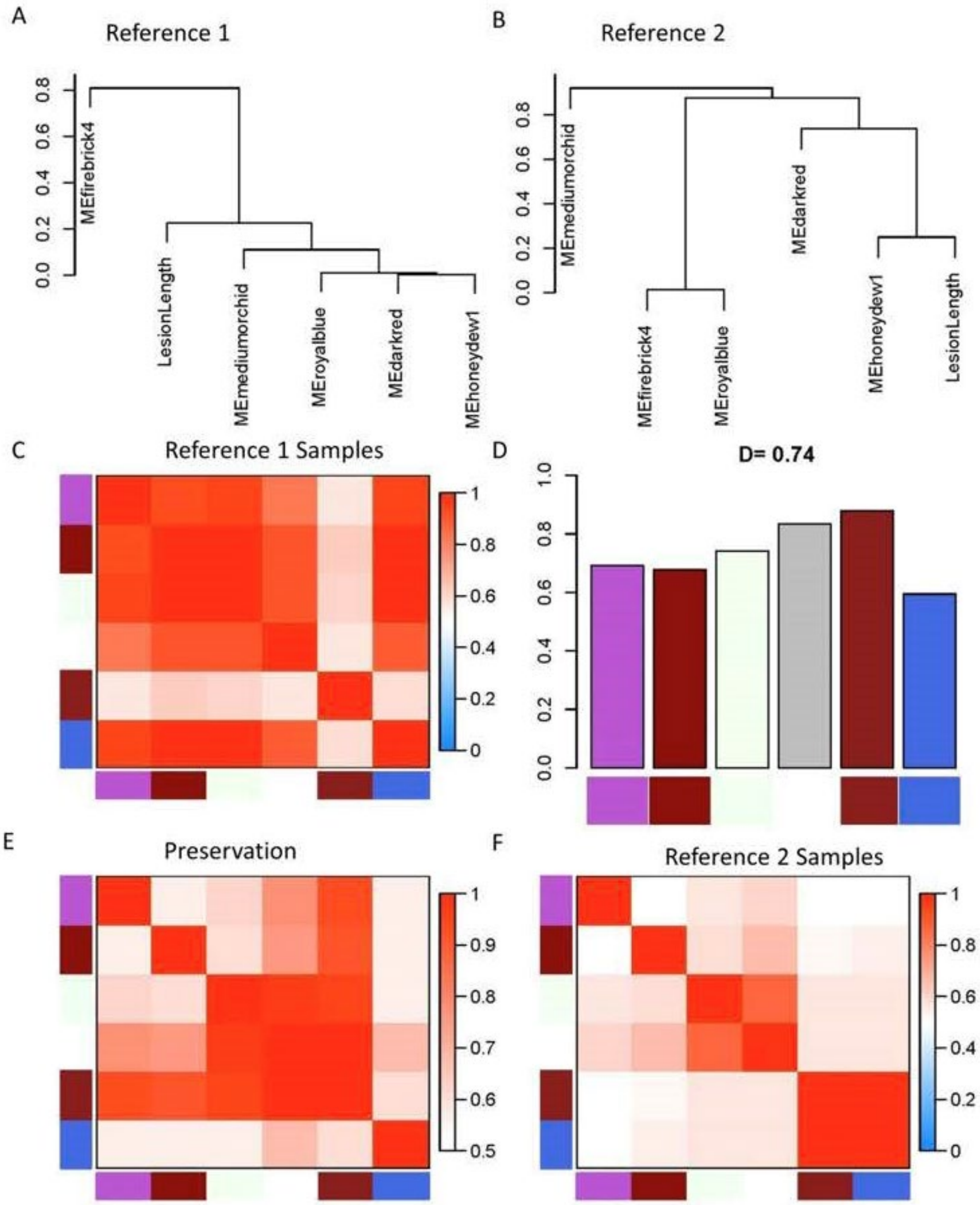

Figure S6. Module preservation of WGCNA reference network 1 and 2

Module preservation between two positively correlated network analyses. A. Hierarchical clustering tree of consensus modules based on reference network 1 module eigengenes. B Hierarchical clustering tree of consensus modules based on reference network 2 module eigengenes. C Heat map of the relationship between the two consensus modules based on adjacency for reference network 1. D Preservation of consensus module eigengenes for reference network 1 and 2. E Heat map of the preservation network, scale indicates adjacency; ranging from 1 (red) high adjacency to 0.5 (white) low adjacency. F Heat map of the relationship between the two consensus modules based on adjacency for reference network 2.

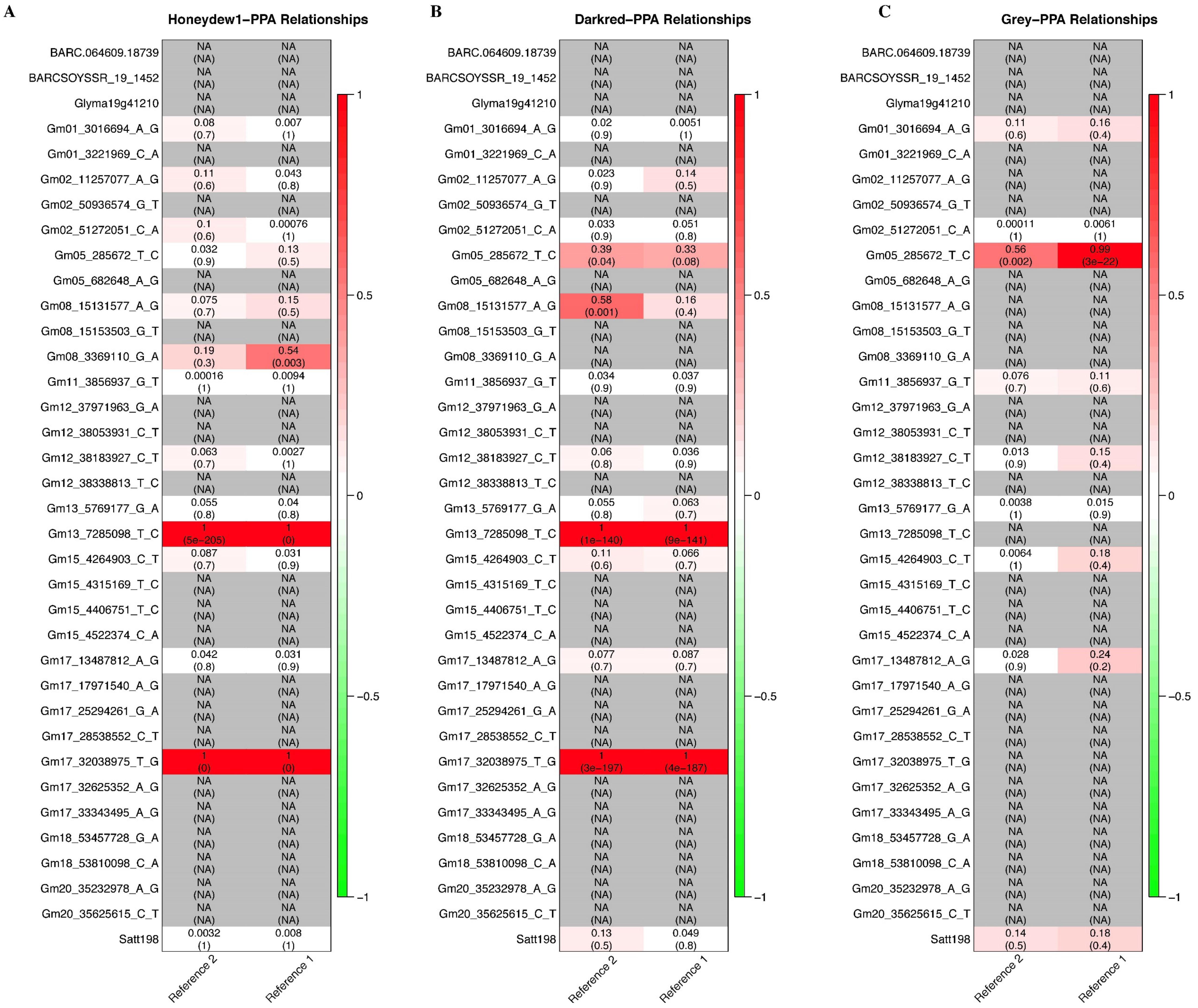

Figure S7. Correlation of Posterior Probability of Association (PPA) values with significant inoculated hotspots

Correlation of PPA values of inoculated eQTL hotspots with genes inside the positively correlated (A) honeydew (B) darkred (C) grey co-expression module. Correlation coefficient scale of 1 to -1, with positive 1 represented by red and negative 1 represented by green. The y-axis represents hotspot SNPs, and x-axis represents two positively correlated reference modules. Top number represents correlation coefficient, number in parenthesis represents P-value < 0.05.
